# Waterlogging and hypoxia in plant abiotic stress

**DOI:** 10.3389/fpls.2026.1927738

**Published:** 2026-09-21

**Authors:** Ashutosh Kumar, Mankesh Kumar, Nitish Kumar, Kanak Saxena, Rabiya Parveen, Digvijay Singh, Suresh Prasad Singh, Satyendra, Rafat Sultana, Sweta Sinha

**Affiliations:** 1 Department of Genetics and Plant Breeding, Bihar Agricultural University, Sabour, Bihar, India; 2 Krishi Vigyan Kendra, Bihar Agricultural University, Sabour, Bihar, India; 3 Department of Seed Science and Technology, Acharya Narendra Deva University of Agriculture and Technology, Kumarganj, Uttar Pradesh, India; 4 Indian Council of Agricultural Research (ICAR)-National Institute of Seed Science and Technology, Mau, Uttar Pradesh, India; 5 School of Drought Stress Management, Indian Council of Agricultural Research (ICAR)-National Institute of Abiotic Stress Management (NIASM), Baramati, India; 6 Department of Molecular Biology and Biotechnology, Bihar Agricultural University, Sabour, Bihar, India

**Keywords:** aerenchyma, anoxia, climate resilience, ERF-VII, fermentation, flooding tolerance, hypoxia, N-degron pathway

## Abstract

Waterlogging is a major abiotic stress affecting 12–27% of global cultivated land and causing average yield losses of 20–33% in cereals and legumes. This poses a serious threat to food security in flood-prone regions. Soil saturation reduces gas diffusion nearly 10,000-fold compared to air. This rapidly induces hypoxia or anoxia in the root zone within hours to days. This oxygen limitation forces a metabolic shift from efficient aerobic respiration (approximately 36 ATP per glucose) to anaerobic fermentation (approximately 2 ATP per glucose). This shift results in severe energy deficits, cytoplasmic acidosis, and accumulation of toxic metabolites such as ethanol, acetaldehyde, and organic acids. Plants exhibit diverse adaptive responses to flooding stress, including morphological modifications such as aerenchyma formation, adventitious root development, hypertrophied lenticels, and shoot elongation in deepwater rice. These modifications facilitate internal oxygen transport and gas exchange. At the biochemical level, fermentative enzymes including alcohol dehydrogenase and pyruvate decarboxylase are upregulated, while antioxidant defense systems mitigate oxidative damage during reoxygenation. Hormonal crosstalk among ethylene, abscisic acid, auxin, and gibberellins regulates the balance between escape and quiescence strategies. At the molecular level, oxygen sensing is primarily mediated by the N-degron pathway through Group VII Ethylene Response Factors (ERF-VIIs). Plant cysteine oxidases regulate oxygen-dependent degradation under normoxia, while hypoxia stabilizes ERF-VIIs to activate anaerobic gene expression. Nitric oxide further modulates ERF-VII proteolysis and stability. The *Sub1A* locus in rice exemplifies genetic adaptation, conferring 14–18 days of submergence tolerance and significant yield advantages in flood-prone environments. Despite these advances, major crops such as wheat, maize, and soybean remain highly susceptible, underscoring the need for translating mechanistic insights into field-level solutions. Integrating CRISPR/Cas-based gene editing, genomic selection, multi-omics approaches, and improved agronomic practices offers promising avenues for developing flood-resilient crop varieties under changing climatic conditions.

## 1 Introduction

Waterlogging occurs when excess water saturates the soil, displacing oxygen and creating low-oxygen conditions around plant roots (Fukao et al., 2019; Zhang et al., 2025). In common usage, waterlogging refers to soil saturation with only the roots submerged, whereas submergence describes water covering the entire plant canopy (Fukao et al., 2019; Zhang et al., 2025). Flooding is a broader term encompassing both conditions, often caused by heavy rainfall or overflowing waterways. Under flooding, root respiration shifts from aerobic to anaerobic, stomata close, chlorophyll degrades, and both photosynthesis and nutrient uptake decline sharply (Zhang et al., 2025). In severe cases, prolonged hypoxia causes root and shoot death, leading to crop failure. The severity of waterlogging stress depends on saturation depth and duration: events lasting fewer than 3–6 days cause minor yield losses, while saturation exceeding two weeks can reduce yields by 50% or more (Zhang et al., 2025; Tian et al., 2021).

Waterlogging and flooding are widespread agricultural challenges. Approximately 12% of global arable land faces regular waterlogging, resulting in average yield reductions of around 20% (Pandey et al., 2010; Chhabra et al., 2025). Annual flooding affects roughly 27% of cultivated land, causing tens of billions of dollars in damage (Liu et al., 2023). A global meta-analysis found that waterlogging reduces average crop yields by about 33% compared to well-drained conditions, with losses during reproductive stages typically exceeding those during vegetative stages, reaching around 42% at flowering versus 35% during vegetative stages (Tian et al., 2021).

### 1.1 Increasing incidence due to climate change

Climate change is intensifying waterlogging risks. Rising temperatures accelerate the hydrological cycle, increasing the frequency and intensity of rainstorms and floods (Zhang et al., 2025; Liu et al., 2023). Flood-prone regions such as monsoon Asia and floodplains worldwide face greater exposure to waterlogged soils. As a result, climate models project that yield losses from waterlogging could double by 2080 under current emission trajectories, rising from 3–11% in the late twentieth century to 10–20% in crop growth projections (Liu et al., 2023). These figures come from global crop models and carry real uncertainty. Actual losses will vary widely by region, crop, soil type, and how much farmers and governments invest in drainage and adaptation, so the numbers are best read as an indication of direction and scale rather than a precise forecast. Annual flood-related crop damages worldwide have reached approximately $19 billion, a figure that has climbed over the past half-century (Liu et al., 2023). Severe annual flood events cause losses of around 18 million tonnes of rice, roughly 4.3% of global rice yield, with China and India losing approximately 10 million and 5 million tonnes per year respectively (Li et al., 2025b). These figures highlight the urgent need for flood-tolerant crop varieties and improved drainage infrastructure.

### 1.2 Vulnerable agro-ecological zones: Indo-Gangetic Plains

Low-lying alluvial plains are particularly prone to waterlogging. The Indo-Gangetic Plains (IGP) of South Asia, spanning northern India, Pakistan, Bangladesh, and Nepal, support roughly 13.5 million hectares of rice-wheat cropping and feed nearly 40% of India’s population (Jat et al., 2019). Poor drainage combined with heavy monsoon rainfall renders large parts of the IGP chronically waterlogged. Satellite data show that about 51% of northern Bihar faces high or very high flood-waterlogging risk during monsoon seasons (Pandey et al., 2010). The Ganges and Brahmaputra floodplains experience regular inundation, with large areas of farmland submerged or waterlogged for weeks each monsoon, significantly reducing yields of maize, wheat, rice, and pulses. In response, farmers in the IGP adopt raised-bed planting, improved drainage, and adjusted cropping schedules. Raised-bed planting in rainfed wheat has been shown to substantially improve yields under waterlogged conditions (Chhabra et al., 2025). Climate change poses an increasing threat to the IGP through more intense flooding, making flood-tolerant crop varieties and engineering solutions essential for long-term agricultural stability.

### 1.3 Crop-specific economic losses and responses

Flooding-induced waterlogging causes major yield and economic losses across cereals and legumes. A global meta-analysis reported an average yield reduction of about 33% under waterlogging (Tian et al., 2021). The severity, and the reasons behind it, vary by crop.

*Rice (Oryza sativa)*: Severe floods reduce global rice production by about 4.3% annually, around 18 million tons. Losses have worsened since 2000 due to more frequent extreme weather (Li et al., 2025b). As a semi-aquatic plant, rice tolerates short submergence far better than other cereals. Varieties carrying the *Sub1A* gene survive approximately 14–18 days of complete submergence, though damage becomes more pronounced toward the upper end of this range (Xu et al., 2006; Fukao et al., 2019). Deepwater varieties instead use *SNORKEL* genes. These genes elongate the stem to keep leaves above rising water. Even so, submergence beyond about a week still causes real damage. It causes roughly 50% yield loss even in tolerant rice, and it typically kills non-tolerant plants outright (Li et al., 2025b).

*Wheat (Triticum aestivum)*: Wheat is far less tolerant of waterlogging than rice. Yield losses typically run 25–30% on average (Tian et al., 2021), and they increase with duration. Saturation lasting more than 7–10 days during sensitive stages, such as tillering or heading, causes serious yield penalties (Ding et al., 2020). Brief, well-drained events may keep losses below 20%. Mechanistically, waterlogging reduces photosynthesis and tillering early in the season and causes spike sterility if it occurs later. Raised beds, ridging, and subsurface drainage all help manage these losses in flood-prone areas (Chhabra et al., 2025).

*Maize (Zea mays)*: Maize is highly sensitive to waterlogging, especially during early vegetative stages. At this stage, low oxygen severely limits nutrient uptake and can cause root decay. Even brief flooding of 3–6 days can cut yield by approximately 40% (Tian et al., 2021). Waterlogging at the V3 (three-leaf) stage has been reported to reduce yield by 58–70% in controlled trials (Huang et al., 2022), and longer events of 9–15 days can exceed 70% loss (Tian et al., 2021). Bed planting and rapid drainage are the main management tools in waterlogged soils.

*Legumes (soybean, peanut, pulses):* Waterlogging can reduce soybean seed yield by 40–80% depending on growth stage and flood duration (Adegoye et al., 2023). Peanuts also suffer sharp yield declines under waterlogging (Zeng et al., 2022). Although data on chickpea and lentil are limited, upland pulse crops generally show yield losses comparable to cereals in saturated soils. Legumes generally lag behind cereals in dedicated waterlogging research, a gap noted in recent surveys of abiotic-stress tolerance across both crop groups (Rane et al., 2021).

### 1.4 Agricultural regions prone to waterlogging

Beyond the IGP, waterlogging affects a wide range of agricultural regions globally. Flat alluvial plains with high water tables, such as those found in Bangladesh, the Mekong Delta, sub-Saharan Africa, and river-irrigated plains in China, face regular seasonal flooding. In India alone, approximately 6 million hectares of farmland have historically experienced chronic waterlogging, with waterlogged soils often becoming saline or alkaline over time (Singh, 2017). India’s approximately 58.2 million hectares of wetlands, most concentrated in the IGP, reflect the scale of water-surplus zones where excess monsoon runoff accumulates in fields (Singh, 2017). Intensive groundwater irrigation in Punjab, Haryana, and western Uttar Pradesh has raised water tables further, compounding waterlogging and salinity problems.

### 1.5 Scope of this review

Waterlogging is a growing threat to global agriculture, exposing an estimated 12–27% of farmed land each year, with climate change accelerating the problem (Zhang et al., 2025; Liu et al., 2023). In flood-prone regions like the IGP, crops such as rice, wheat, and maize suffer yield penalties of 20–50% under waterlogged conditions (Li et al., 2025b; Tian et al., 2021). This review examines the physiological, biochemical, molecular, and genetic dimensions of waterlogging and hypoxia tolerance in plants, from oxygen sensing and energy metabolism to morphological adaptation, hormonal regulation, and genomic architecture, with the aim of identifying translational opportunities for crop improvement under changing climatic conditions.

## 2 Concept of hypoxia and anoxia in plant systems

Plants require oxygen as the final electron acceptor in mitochondrial respiration. Oxygen comprises about 21% of air, but even under well-aerated conditions, internal oxygen levels in dense tissues can drop to 1–7% (Colmer and Voesenek, 2009). Hypoxia refers to insufficient oxygen, typically defined as below 1–5% O_2_, while anoxia refers to its complete absence at 0% O_2_ (Jethva et al., 2022; Loreti and Perata, 2020). Acute hypoxia, which develops rapidly during sudden flooding, is generally harmful and treated as stress. Chronic hypoxia, by contrast, can occur naturally in tissues such as the shoot apical meristem and lateral root primordia, where oxygen levels below 5% serve as a developmental signal rather than a stress condition (Loreti and Perata, 2020).

### 2.1 Oxygen thresholds for plant survival

Under normal oxygen conditions of around 21% O_2_, mitochondrial electron transport fully supports ATP production. As oxygen falls below 5%, electron transport decreases progressively (Jethva et al., 2022; van Veen et al., 2024). Many temperate crops show stress when soil or tissue oxygen drops below about 10 kPa (roughly 10% O_2_ at sea level) and die if anoxic conditions persist for hours to days. Rice and flood-adapted species tolerate far lower oxygen levels. Rice coleoptiles maintain energy levels through prolonged anoxia, while wheat seedlings deplete their ATP within 24 h (Magneschi and Perata, 2009). This species-dependence is stark even during germination. Rice extends its coleoptile and continues growth under complete anoxia. Barley and oat, by contrast, fail to germinate under the same conditions (Magneschi and Perata, 2009).

Dense plant organs often develop natural hypoxic microenvironments due to high respiration and low gas diffusion. For example, the core of a growing potato tuber can reach 5% O_2_, and barley seeds can become nearly anoxic internally (Jethva et al., 2022). These naturally low-oxygen zones are not stressful and are in fact necessary for normal development (Loreti and Perata, 2020). Environmental hypoxia caused by flooding, however, is typically sudden and damaging. Oxygen thresholds for survival are therefore species-dependent and better described as a continuum rather than a fixed number.

### 2.2 Aerobic vs. anaerobic metabolism

Under aerobic conditions, glycolysis, the tricarboxylic acid (TCA) cycle, and the mitochondrial electron transport chain, together oxidize glucose completely, producing approximately 36–38 ATP per glucose (the classical theoretical maximum, based on integer P/O ratios) (Magneschi and Perata, 2009). Under hypoxia or anoxia, the electron transport chain is blocked and NAD^*+*^ must be recycled through fermentation. Pyruvate is either reduced to lactate by lactate dehydrogenase or decarboxylated to acetaldehyde by pyruvate decarboxylase and then converted to ethanol by alcohol dehydrogenase, yielding only about 2 ATP per glucose (Magneschi and Perata, 2009; van Veen et al., 2024).

This energy deficit is severe. Anoxia can reduce a plant’s energy output by 65–97% compared to aerobic conditions (Gibbs and Greenway, 2003). Plants respond by downregulating energy-intensive processes such as protein synthesis and plasma membrane ATP pumping, redirecting the limited ATP available toward membrane maintenance and ion balance rather than growth (Magneschi and Perata, 2009; van Veen et al., 2024). The shift from oxidative to fermentative metabolism is therefore the fundamental biochemical stress of hypoxia, characterized by limited energy, toxic by-products, and eventual energy starvation unless oxygen is restored.

### 2.3 Soil physics and oxygen deprivation

#### 2.3.1 Soil pore saturation and gas diffusion

When soil pores fill with water, air-filled spaces collapse and gas exchange becomes very difficult. Oxygen moves through water approximately 10,000 times more slowly than through air (Currie, 1965), so saturated soils quickly trap respiratory gases and block the influx of atmospheric oxygen (Bailey-Serres et al., 2012). Microbial and root respiration rapidly consume remaining oxygen, pushing the soil into hypoxia. The oxygen diffusion coefficient in saturated soil drops sharply, effectively making waterlogged soils anaerobic environments where aerobic respiration cannot occur (Bailey-Serres et al., 2012; Zhang et al., 2025).

#### 2.3.2 Transition from aerobic to anaerobic soil conditions

Once diffusion is blocked, remaining oxygen in pore water is consumed by roots and microbes. Aerobic metabolism stops and anaerobic processes take over within hours to days of flooding (Fukao et al., 2011). ATP production in plants drops sharply, while oxygen levels in the root zone remain near zero (Gibbs and Greenway, 2003; Fukao et al., 2011). Although root aerenchyma can help maintain internal aeration in well-aerated soils, these adaptations generally fail to prevent hypoxia in fully flooded conditions (Yamauchi et al., 2018).

#### 2.3.3 Redox potential changes under waterlogging

Anaerobic soils show a sharp drop in redox potential (Eh). Microbes sequentially use alternative electron acceptors as oxygen is depleted, following the order: nitrate → manganese → iron → sulfate → carbon dioxide (methanogenesis) (Ponnamperuma, 1984). Soil Eh in flooded areas can fall from above +400 mV under aerobic conditions to below −300 mV, driving these sequential redox reactions. These are typical ranges rather than fixed cutoffs. In practice the transitions form a continuum, and the Eh at which each process dominates shifts with soil pH, temperature, and organic matter content. As Fe(III) and Mn(IV) are reduced to Fe^2+^ and Mn^2+^, these ions become soluble and potentially toxic in the root zone (Colmer and Voesenek, 2009).

#### 2.3.4 Formation of toxic reduced compounds

Anaerobic soil chemistry produces several harmful by-products. Sulfate reduction generates hydrogen sulfide (H_2_S), a toxin that migrates toward roots. Fermentation products including ethanol, lactate, acetic acid, and butyric acid accumulate under hypoxia, acidifying the rhizosphere and impairing root function (Bailey-Serres et al., 2012). Under extended anoxia, methanogenic archaea produce methane from available carbon (Ponnamperuma, 1984). Together, Fe^2+^, Mn^2+^, H_2_S, organic acids, and methane create a chemically hostile root environment, as shown in **Figure 1**, leading to the physiological decline of most upland crops during flooding (Herzog et al., 2016).

**Figure 1:**
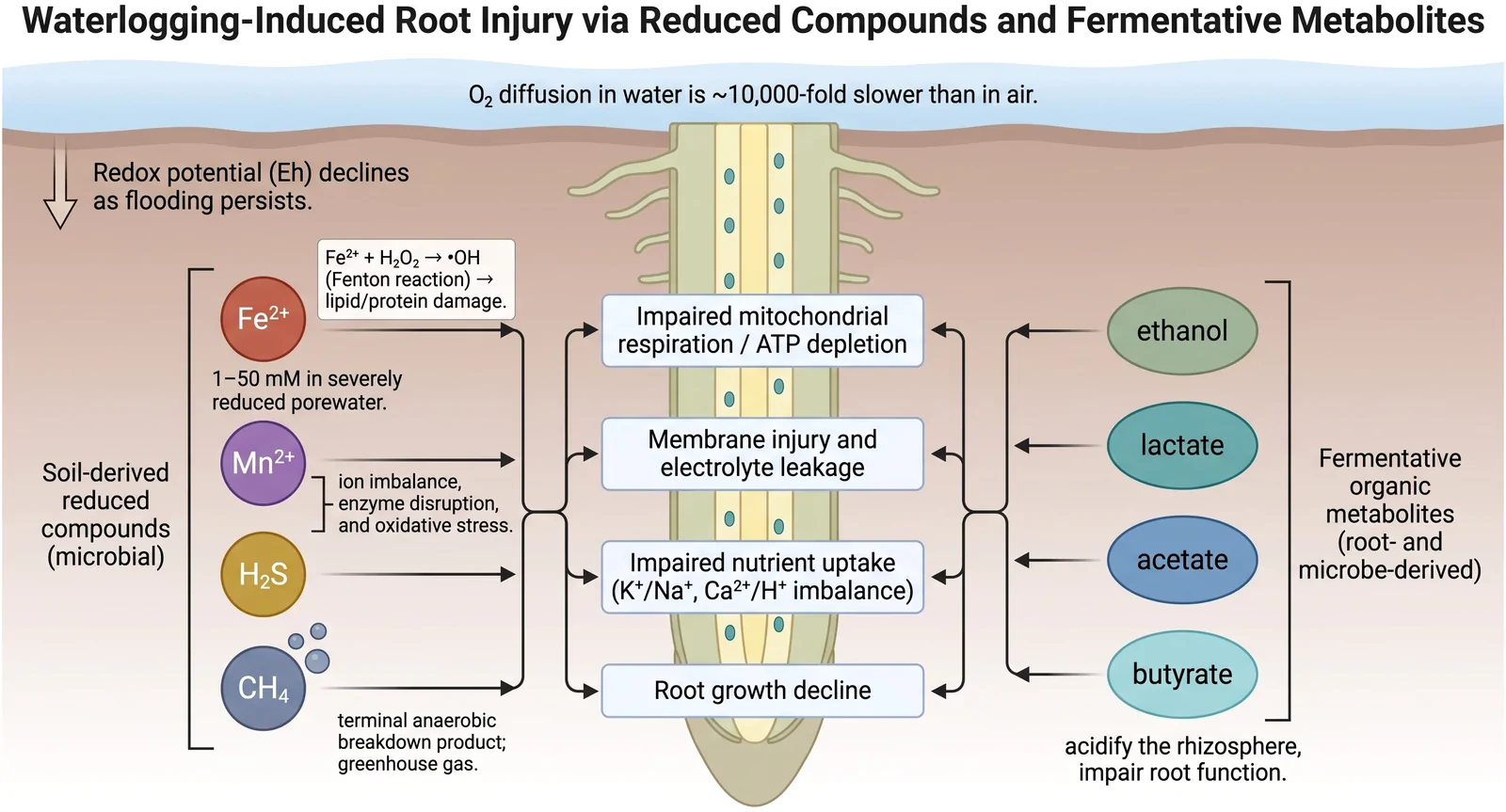
Reduced compounds and metabolites produced in waterlogged soils and their effects on roots. The sequential redox reactions and associated root-zone effects are detailed in **Figure 4**. Based on Ponnamperuma (1984) and Herzog et al. (2016).

## 3 Hypoxia and anoxia

Agriculture faces two persistent challenges: feeding a growing global population and maintaining stable food production under unpredictable climate conditions. Flooding and waterlogging together affect roughly 27% of farmland worldwide each year, causing economic losses in the tens of billions of dollars (Liu et al., 2023). While drought harms plants through dehydration, flooding damages them by cutting off their oxygen supply. As detailed in Section 2.3, soil gas diffusion collapses under these conditions. As a result, plant roots can be almost completely deprived of oxygen within hours of waterlogging (Colmer and Voesenek, 2009).

Plants respond to flooding across multiple time scales. Within seconds of oxygen depletion, calcium signals trigger phosphorylation cascades. Within minutes, the N-degron oxygen-sensing pathway stabilizes Group VII Ethylene Response Factor (ERF-VII) transcription factors, which activate the core anaerobic gene program. Within hours, glycolysis shifts toward alcohol fermentation. Within days, aerenchyma forms through programmed cell death in root cortical cells. Over weeks, hormonal signals promote adventitious root growth and lenticel enlargement, leading to either stem elongation or developmental quiescence depending on the plant’s genetic makeup (Vartapetian and Jackson, 1997). These multilevel responses are integrated across molecular, physiological, and morphological scales, as illustrated in **Figure 2**.

**Figure 2:**
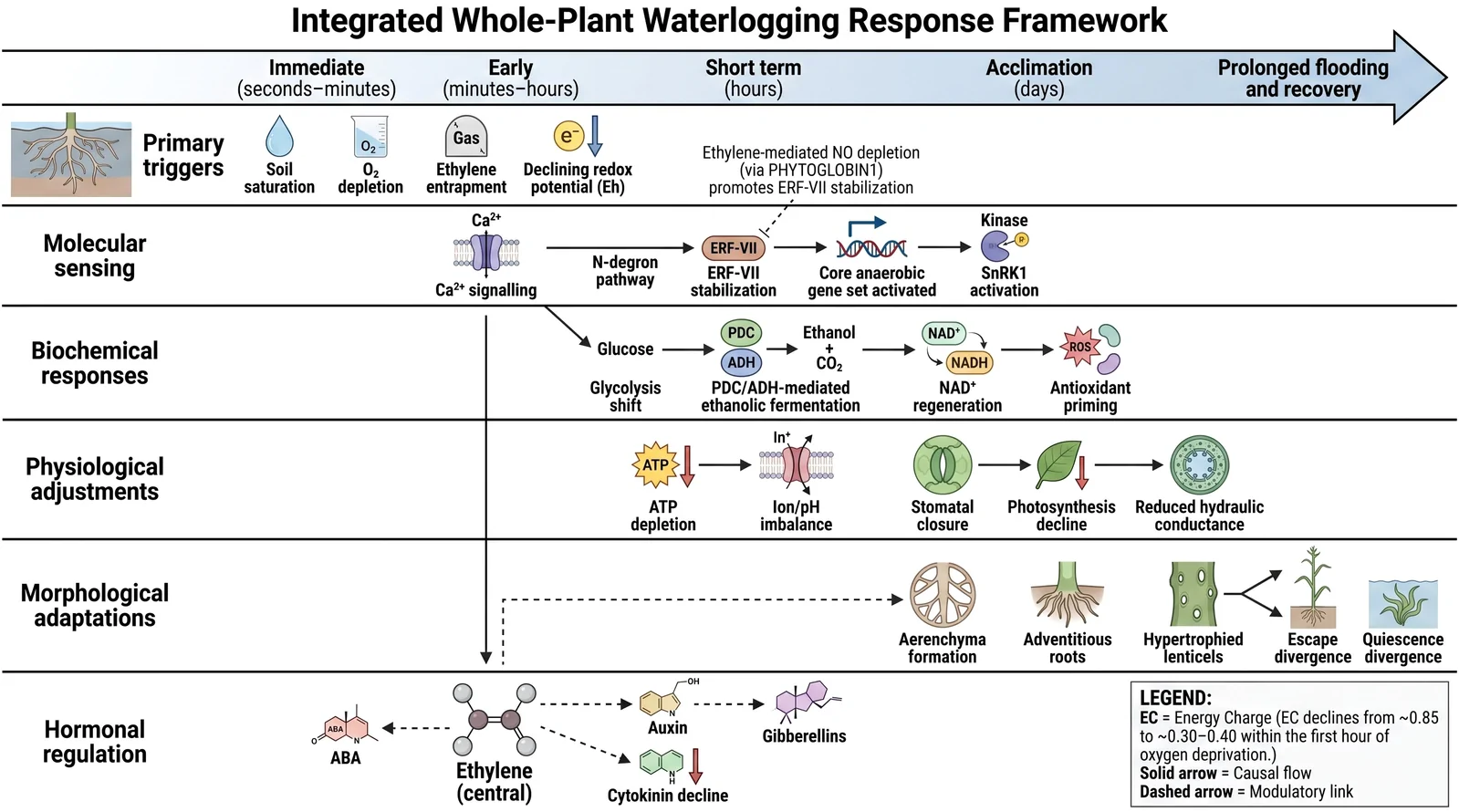
Integrated whole-plant waterlogging response framework. Six tiers (rows) represent primary triggers, molecular sensing, biochemical responses, physiological adjustments, morphological adaptations, and hormonal regulation. Five response categories (columns) operate simultaneously across the flooding timeline. Each cell summarizes key processes and representative molecules. Synthesized from Bailey-Serres and Voesenek (2008) and Voesenek and Bailey-Serres (2015).

The discovery of the N-degron oxygen-sensing pathway in 2011 provided the first mechanistic explanation of how plants perceive and respond to cellular oxygen depletion at the protein level (Gibbs et al., 2011; Licausi et al., 2011). Subsequent research established that ethylene-mediated nitric oxide depletion promotes ERF-VII stabilization, enabling pre-adaptive responses before the onset of severe hypoxia (Hartman et al., 2019). Genome-wide translatome profiling and gene regulatory network mapping have since characterized the hypoxia-responsive transcriptional landscape at single cell-type resolution (Mustroph et al., 2010; Reynoso et al., 2019). At the applied level, introgression of the *Sub1A* locus into high-yielding rice cultivars has substantially improved submergence tolerance for smallholder farmers across flood-prone regions of Asia (Septiningsih et al., 2009).

This review covers the full range of plant responses to flooding stress, from oxygen sensing and cellular biochemistry to whole-plant physiology, organ-level adaptations, hormonal regulation, and genomic architecture. Discoveries made after 2019 are incorporated throughout to reflect the current state of the field. The review concludes by evaluating research priorities and breeding strategies needed to develop flood-resilient varieties for a changing climate (Bailey-Serres et al., 2019).

### 3.1 Definitions: hypoxia and anoxia

As established in Section 2, hypoxia and anoxia form a continuum of oxygen deficiency rather than two fixed states. At the mitochondrial level, though, this continuum has a precise biochemical endpoint. Under true anoxia, oxidative phosphorylation ceases entirely, because Complex IV is fully deprived of its terminal electron acceptor (Millar et al., 2011).

In agricultural settings, waterlogging typically produces hypoxia rather than strict anoxia in root tissues, as trace oxygen is maintained by residual gas pockets and restricted diffusion through the water film. True anoxia occurs only in deeply compacted flooded soils or in the core of large root organs where aerenchyma-mediated oxygen supply is insufficient to meet respiratory demand (Gibbs and Greenway, 2003). The N-degron pathway, mediated by plant cysteine oxidases (PCO1–PCO5), operates across physiologically relevant hypoxic oxygen concentrations of 0.1–5%, providing a graded concentration-dependent response rather than a binary on/off switch (Voesenek and Bailey-Serres, 2015).

### 3.2 Role of mitochondria and energy crisis under hypoxia

The mitochondrion is the primary site of oxygen consumption in cells and the first organelle to be impaired when oxygen supply falls below critical levels. Under normoxia, the electron transport chain moves electrons from NADH and FADH_2_ through Complexes I, II, III, and IV, reducing O_2_ to water. This process drives ATP synthase through a proton-motive force, generating approximately 30–32 mol ATP per mol glucose, a more precise estimate than the classical 36–38 figure in Section 2.2, based on measured, non-integer P/O ratios (Millar et al., 2011). Although Complex IV has a low K*m* for O_2_ of roughly 0.1–1.0 μM, giving it a high affinity for oxygen, ATP synthesis declines once tissue oxygen falls below the threshold needed to sustain electron transport.

Oxygen limitation imposes an immediate energy penalty on root cells. The cellular energy charge is maintained near 0.85 under well-oxygenated conditions but falls sharply to 0.30–0.40 within the first hour of oxygen deprivation (Saglio et al., 1983). This energy collapse disrupts virtually every ATP-dependent process in the cell. One of the earliest casualties is the plasma membrane H^+^-ATPase, which relies on ATP to pump protons and maintain electrical balance across root cell membranes. When ATP falls, this pump slows, the membrane depolarizes, and ion balance across K^+^/Na^+^ and Ca^2+^/H^+^ gradients breaks down, impairing active nutrient absorption (Felle, 2005).

This bioenergetic crisis triggers activation of SnRK1 (sucrose non-fermenting-1-related kinase 1), the plant cell’s central energy-sensing kinase. Section 3.3.4 details its trehalose-6-phosphate (T6P)-dependent activation mechanism and downstream targets, together with the accompanying cytoplasmic acidosis. That section treats SnRK1 signaling as part of the broader molecular oxygen-sensing network.

### 3.3 Molecular basis of oxygen sensing

#### 3.3.1 The N-degron pathway: how plants detect falling oxygen

In 2011, two independent research groups simultaneously identified the molecular mechanism by which plants sense cellular oxygen concentration. Both teams identified a branch of the ubiquitin-proteasome system, known as the N-degron pathway, as the primary oxygen sensor (Gibbs et al., 2011; Licausi et al., 2011). Plant cysteine oxidases (PCO1–PCO5 in *Arabidopsis thaliana*) oxidize the N-terminal cysteine residues of ERF-VII transcription factors to cysteine sulfinic acid, consuming one molecule of O_2_ per catalytic cycle (Weits et al., 2014). Because PCO activity depends on molecular oxygen as a co-substrate, these enzymes act as sensitive, graded, and reversible oxygen sensors. The detailed mechanism of this pathway is shown in **Figure 3**.

**Figure 3:**
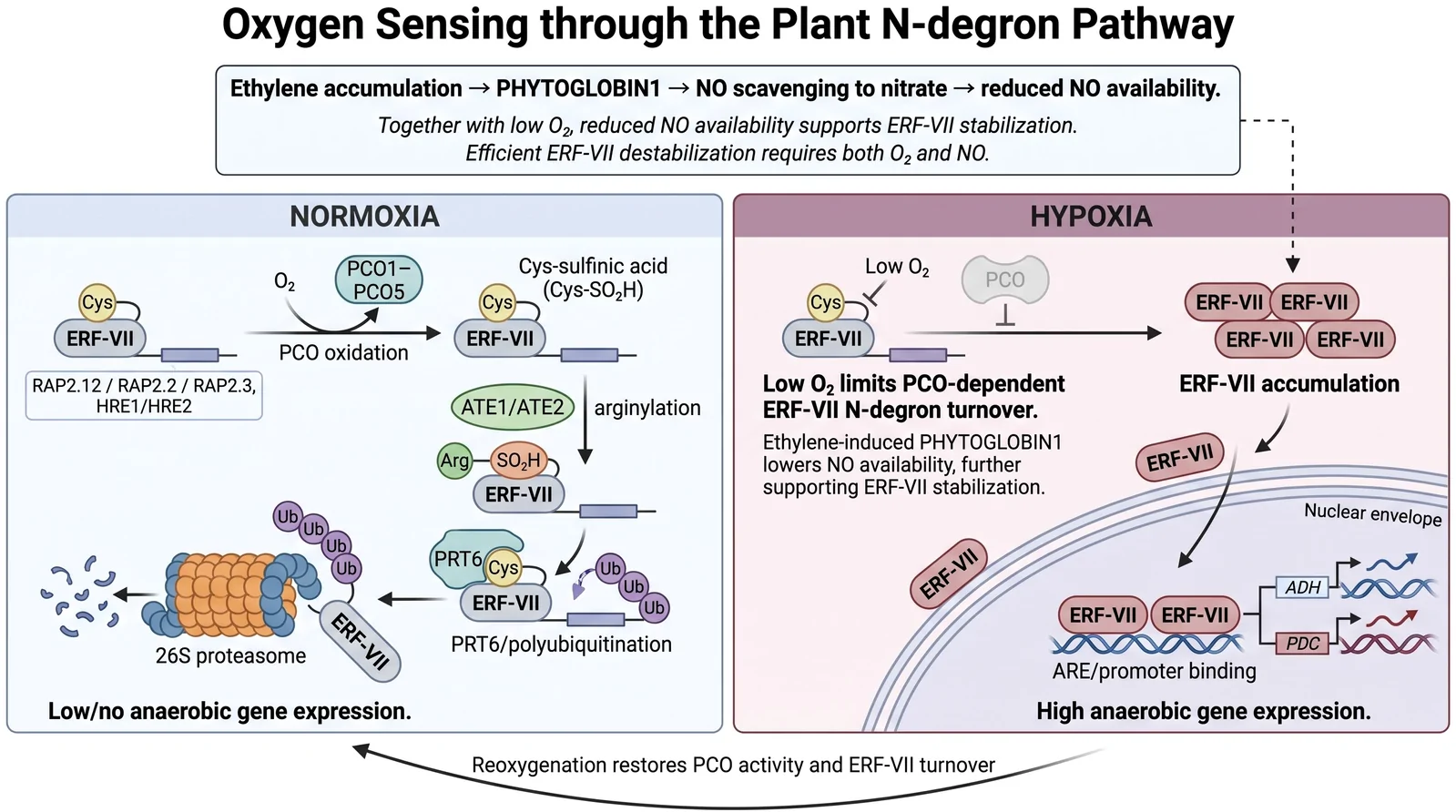
N-degron pathway. Under normoxia (left), Plant Cysteine Oxidases (PCOs) oxidize the N-terminal Cys of ERF-VII to Cys-SO_2_H; ATE1/ATE2 arginylation and PRT6 ubiquitination direct rapid 26S proteasomal degradation. Under hypoxia, PCO suppression stabilizes ERF-VII, enabling nuclear translocation and activation of hypoxia-responsive genes. Ethylene-mediated NO depletion promotes ERF-VII stabilization before severe hypoxia develops (Gibbs et al., 2014; Hartman et al., 2019). Based on Gibbs et al. (2011); Licausi et al. (2011); Weits et al. (2014).

Under normoxic conditions, PCO enzymes oxidize the N-terminal cysteine of ERF-VII substrates, which is recognized by arginyl-tRNA transferases ATE1 and ATE2. These enzymes append an arginine residue to the modified terminus, which is then recognized by the E3 ubiquitin ligase PRT6, directing polyubiquitination and rapid 26S proteasomal degradation, often within minutes of ERF-VII synthesis (Varshavsky, 2019). Under hypoxic conditions, declining PCO activity reduces N-terminal cysteine oxidation, blocking the downstream degradation cascade. Stabilized ERF-VII proteins accumulate, translocate to the nucleus, and bind Anaerobic Response Elements in the promoters of hypoxia-responsive genes, activating the core anaerobic gene program (Weits et al., 2014). A defining feature of this system is its full reversibility: upon reoxygenation, PCO activity is restored, the degradation cascade is reactivated, and ERF-VII proteins are rapidly cleared (Gibbs et al., 2011; Licausi et al., 2011).

#### 3.3.2 Nitric oxide in ERF-VII proteolytic regulation

Nitric oxide (NO), together with oxygen, contributes to the proteolytic regulation of ERF-VII transcription factors. During early flooding, ethylene accumulation induces PHYTOGLOBIN1-mediated NO scavenging, thereby promoting ERF-VII stabilization before severe hypoxia develops (Gibbs et al., 2014; Hartman et al., 2019). 

How independent these two inputs really are, though, is still being worked out. Biochemical evidence shows that efficient destabilization of ERF-VII requires both oxygen and NO acting together. A drop in either gas alone can be enough to stabilize the protein (Gibbs et al., 2014). This points to a shared reaction that needs both gases, rather than two fully separate sensing routes. Exactly how much each gas contributes under natural, fluctuating field conditions, as opposed to controlled lab conditions, remains an open question.

This same NO-depletion mechanism operates through a dedicated scavenging system. Class 1 phytoglobins, also known as class 1 hemoglobins, scavenge NO rather than generate it. They convert it to nitrate through a dioxygenase reaction, lowering cellular NO availability and thereby favoring ERF-VII stabilization. Phytoglobin 1 expression rises under hypoxia, and its NO-scavenging activity ties oxygen sensing, nitric oxide balance, and cellular redox status together into one regulatory network rather than three separate pathways (van der Wijk et al., 2026). This scavenging role means NO signaling during flooding depends not only on how much is produced but on how quickly it is removed, adding a further layer to the uncertainty already noted above.

Weits et al. (2019) separately showed that the N-degron pathway also controls a shoot-meristem-localized hypoxic niche via the substrate ZPR2, extending N-degron-mediated oxygen sensing beyond flooding responses into normal developmental regulation.

#### 3.3.3 Group VII ERF transcription factors: diversity and downstream targets

The ERF-VII family in *Arabidopsis* has five members, and rice has several functional equivalents; **Table 1** summarizes each gene’s functional domain and mutant or transgenic phenotype. Under normoxia, RAP2.12 and RAP2.2 are sequestered at the plasma membrane through interaction with acylated acyl-CoA-binding protein (ACBP) partner proteins, creating a pool of pre-synthesized ERF-VII proteins that can be released and stabilized within minutes when oxygen drops, without requiring new transcription (Licausi et al., 2011).

**Table 1:** Group VII ERF transcription factors in *Arabidopsis* and their rice functional equivalents: domains and loss/gain-of-function phenotypes.

Species	Gene(s)	Functional domain/regulation	Mutant or transgenic phenotype
*Arabidopsis*	*RAP2.12*	N-degron motif; AP2 domain; constitutively expressed, membrane-tethered via ACBP	Stabilized (Δ13) form alone triggers the full hypoxia response; *rap2.2 rap2.12* double mutant impairs it (Bui et al., 2015)
*Arabidopsis*	*RAP2.2*	N-degron motif; AP2 domain; constitutively expressed, redundant with *RAP2.12*	*rap2.2 rap2.12* double mutant shows hampered hypoxia response (Bui et al., 2015)
*Arabidopsis*	*RAP2.3*	N-degron motif; AP2 domain; overlapping expression with *RAP2.2/RAP2.12*	Contributes to redundant activation of the core hypoxia response (Bui et al., 2015)
*Arabidopsis*	*HRE1*	N-degron motif; AP2 domain; hypoxia-induced transcriptionally	Overexpression improves anoxia tolerance; *hre1 hre2* double mutant more susceptible than wild type (Licausi et al., 2010)
*Arabidopsis*	*HRE2*	N-degron motif; AP2 domain; hypoxia-induced transcriptionally	*hre1 hre2* double mutant more susceptible than wild type; weaker effect than *HRE1* alone (Licausi et al., 2010)
Rice	*Sub1A*	N-degron motif; AP2 domain; part of *Sub1* QTL, chromosome 9	Confers submergence tolerance via quiescence; introgression into intolerant backgrounds confers tolerance (Xu et al., 2006; Fukao et al., 2006)
Rice	*Sub1B, Sub1C*	N-degron motif; AP2 domain; same *Sub1* locus	Contribute less to tolerance than *Sub1A* (Xu et al., 2006; Fukao et al., 2006)
Rice	*SK1, SK2*	N-degron motif; AP2 domain	Drive gibberellin (GA)-mediated internode elongation (escape strategy); opposite strategy to *Sub1A* (Hattori et al., 2009)

Cell-type-specific translatome profiling has revealed extensive hypoxia-responsive translational reprogramming across *Arabidopsis* root cell types (Mustroph et al., 2009). Comparative studies also indicate that several core metabolic and transcriptional responses to low oxygen are conserved across plant species (Mustroph et al., 2010; Narsai et al., 2011). This core regulon was defined using bulk, cell-type-sorted profiling methods, which average gene expression across many cells of the same type. More recent single-nuclei sequencing tells a more detailed story. It shows that hypoxic transcriptional responses vary sharply even within a single root: meristematic cells mount the strongest rewiring, while the vulnerable quiescent center responds quite differently (Hill et al., 2026). How much of ERF-VII stabilization itself is uniform across cell types, rather than just its downstream targets, is still an open question.

#### 3.3.4 Cellular energy crisis and SnRK1-mediated metabolic depression

The biophysical consequences of mitochondrial oxygen sensitivity, including membrane depolarization and ion gradient collapse, are underpinned by a coordinated molecular response centered on SnRK1-mediated metabolic reprogramming. The sharp decline in cellular energy charge triggers signaling cascades that act as early molecular messengers bridging the energy deficit with the cell’s adaptive response (Millar et al., 2011). As flooding reduces sucrose delivery to roots, T6P concentrations decline, releasing SnRK1 from inhibition. SnRK1 translates the decline in cellular energy into coordinated suppression of anabolic metabolism by phosphorylating and inactivating sucrose phosphate synthase, nitrate reductase, and HMG-CoA reductase, substantially reducing biosynthetic ATP demand (Gibbs and Greenway, 2003). This metabolic depression response is more pronounced in waterlogging-tolerant genotypes than in sensitive ones, identifying SnRK1 signaling capacity as a molecular determinant of flooding survival. Cytoplasmic acidosis from lactic acid fermentation further shifts the kinetic balance from lactate dehydrogenase toward pyruvate decarboxylase, limiting further acidification while directing carbon flux toward the more sustainable ethanolic fermentation pathway (Felle, 2005).

### 3.4 Rhizosphere microbial dynamics under waterlogging

This internal energy crisis does not stay contained within the plant. As root cells shift toward fermentation, their oxygen consumption drops, and less oxygen diffuses outward from the root into the surrounding soil. This decline in radial oxygen loss removes the main force that had kept the rhizosphere aerobic. This decline, together with restricted gas diffusion and microbial oxygen consumption after flooding, drives the shift in microbial communities described below.

#### 3.4.1 Sequential reduction and shift to anaerobic microbes

Soil waterlogging triggers a profound restructuring of the rhizosphere microbial community. As oxygen is consumed and soil redox potential declines progressively, a thermodynamically ordered succession of microbial guilds replaces the aerobic community, following the Gibbs free energy hierarchy of available redox couples (Ponnamperuma, 1984). Denitrifying bacteria including *Pseudomonas* and *Paracoccus*, are first to proliferate, reducing nitrate to nitrous oxide and dinitrogen. This nitrogen loss is permanent and agronomically significant, as gaseous N_2_ and N_2_O are unavailable for plant uptake within the cropping season. As Eh continues to decline, iron-reducing bacteria notably *Geobacter* and *Shewanella*, reduce Fe^3+^ to Fe^2+^, driving dissolved Fe^2+^ concentrations in soil porewater to 1–50 mM and generating genuine phytotoxicity. Subsequently, sulfate-reducing bacteria convert sulfate to hydrogen sulfide, which directly inhibits mitochondrial Complex IV. At the lowest redox potentials, methanogenic archaea produce methane as the terminal product of anaerobic organic matter breakdown (Ponnamperuma, 1984). The sequential redox reactions and their plant impacts are summarized in **Figure 4**.

**Figure 4:**
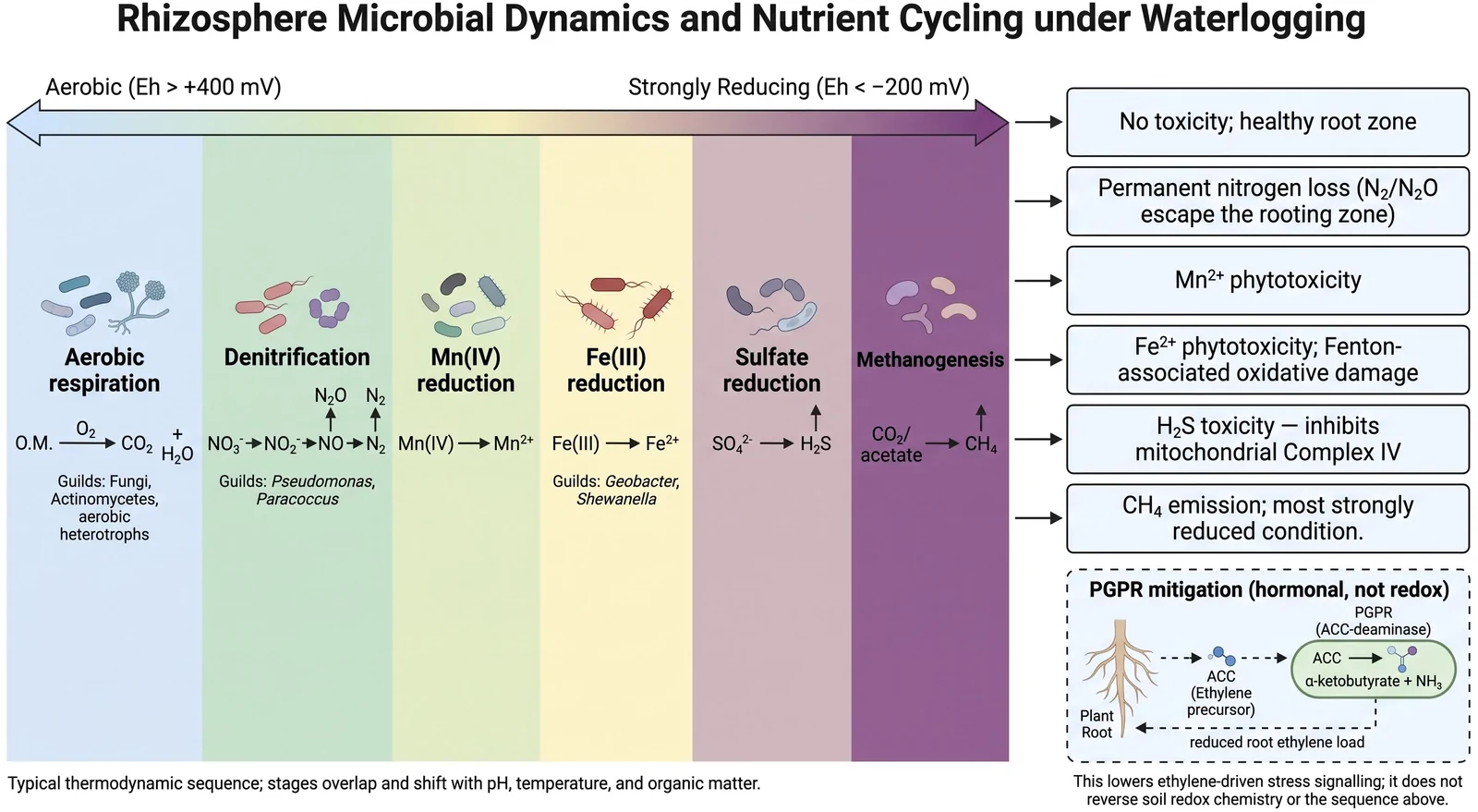
Rhizosphere microbial dynamics and nutrient cycling. Left: sequential redox reduction cascade from aerobic (Eh >+400 mV) through denitrification (NO_3_^−^ → N_2_), Mn(IV) reduction, Fe(III) reduction (increased soluble Fe^2+^), sulfate reduction (H_2_S), to methanogenesis (Eh <–200 mV). The Eh axis is continuous and these stage boundaries overlap and shift with soil pH, temperature, and organic matter content. Right: resulting plant impacts including permanent N loss via denitrification, Fe^2+^/Mn^2+^/H_2_S phytotoxicities, and PGPR mitigation via ACC-deaminase reducing root ethylene accumulation. Based on Ponnamperuma (1984); Becker and Asch (2005); Glick (2014).

Among rhizosphere microorganisms, plant growth-promoting rhizobacteria (PGPR) that produce 1-aminocyclopropane-1-carboxylic acid (ACC) deaminase are well characterized for their capacity to moderate flooding stress. These bacteria degrade ACC, the immediate biosynthetic precursor to ethylene, into alpha-ketobutyrate and ammonia, reducing the hormonal load in root tissues and attenuating downstream flooding-stress responses. Because ACC is a universal intermediate in ethylene biosynthesis across all higher plants, this strategy is not restricted to species-specific signaling architectures and can be deployed alongside conventional breeding programs across diverse crop systems (Glick, 2014).

Field performance is less predictable than these laboratory results suggest, however. A 2026 review of PGPR-based biofertilizers describes a persistent translational gap between well-understood laboratory mechanisms and inconsistent field efficacy, driven by poor rhizosphere colonization, context-dependent gene regulation, and unstable formulations (Wang and Xu, 2026). For ACC-deaminase PGPR specifically, colonization success and ethylene-lowering activity both depend heavily on soil type, native microbial competition, and the specific crop-strain pairing used. Field trial results should therefore be interpreted with this variability in mind.

#### 3.4.2 Impact on nutrient cycling

Waterlogging unravels the biogeochemical cycles that plant roots depend on for basic nutrition. Nitrogen availability is among the most severely affected parameters. Denitrifying bacteria convert plant-available nitrate to gaseous N_2_O and N_2_, lost irreversibly from the rooting zone. Simultaneously, biological nitrogen fixation by symbiotic *Rhizobium* and free-living genera is substantially inhibited in anaerobic environments, curtailing replenishment of inorganic nitrogen reserves. The resulting constraints on the plant's own nitrate assimilation machinery are detailed in Section 4.4.1.

Phosphorus dynamics under waterlogging are more complex. Anaerobic decomposition transiently releases inorganic phosphate into solution, but the concurrent rise in dissolved Fe^2+^ causes ferrous iron to form insoluble complexes with phosphate. As soil redox chemistry fluctuates during and after flooding, these complexes reprecipitate and rapidly withdraw phosphorus from solution, resulting in an erratic phosphate supply throughout the flooding period (Ponnamperuma, 1984). Manganese undergoes an analogous biogeochemical mobilization under these reducing conditions. Its physiological consequences for the plant are detailed in Section 4.4.2.

## 4 Physiological responses to waterlogging

### 4.1 Reduced root respiration and ATP depletion

Roots are the most directly affected tissue during flooding, being the first to experience anaerobic stress and the slowest to recover. Oxygen depletion rapidly collapses the cellular energy charge, disrupting ATP-dependent processes across root cells (Plaxton and Podesta, 2006), the same energy-charge decline detailed in Section 3.2. Beyond limiting respiration, flooding impairs membrane function. As ATP levels fall, plasma membrane H^+^-ATPase activity declines, causing membrane depolarization and breakdown of K^+^/Na^+^ and Ca^2+^/H^+^ gradients, resulting in electrolyte leakage and reduced capacity for nutrient uptake even when nutrients are available in the surrounding soil. Cytoplasmic acidification driven by lactic acid fermentation further impairs pH-sensitive enzymes, creating a self-reinforcing crisis where the tools needed for adaptation are undermined by the very conditions they must address (Felle, 2005).

Across species, K^+^ retention under depolarized anaerobic conditions is consistently identified as a key marker of flooding tolerance. Plants that maintain intracellular potassium under membrane stress show greater survival during prolonged waterlogging, reflecting a broader capacity to preserve membrane integrity and ion regulation (Voesenek and Bailey-Serres, 2015).

### 4.2 Photosynthetic inhibition

While waterlogging primarily damages root tissues, photosynthesis in above-ground shoots is significantly hindered through secondary physiological effects. Flooding lowers root hydraulic conductance by causing acidosis-related dephosphorylation and inactivation of Plasma membrane Intrinsic Protein (PIP) aquaporin channels. This reduces water delivery to leaves despite abundant soil moisture and induces a water-stress-like stomatal closure response. Abscisic acid (ABA) signals reaching leaves via the xylem, together with locally regulated leaf ABA, can activate the PYR/PYL–PP2C–SnRK2–SLAC1 guard-cell signaling pathway, reducing stomatal conductance and intercellular CO_2_ concentration independently of leaf water status (Cutler et al., 2010).

Alongside stomatal closure, ethylene accumulation and reduced cytokinin export from hypoxic roots promote leaf senescence and chlorophyll loss (Voesenek and Bailey-Serres, 2015). The simultaneous depletion of cytokinin export from hypoxic roots removes the main hormonal signal against leaf senescence, releasing the ethylene-driven chlorophyll degradation program. The combined reduction in stomatal conductance, CO_2_ assimilation, and chlorophyll content creates a carbon shortage at precisely the moment when submerged roots most need imported photosynthate for fermentative glycolysis (Voesenek and Bailey-Serres, 2015).

### 4.3 Rhizosphere chemistry and phytotoxin accumulation

The sequential microbial reduction cascade generates phytotoxic compounds that impose direct chemical stress on root tissues. Fe^2+^ and Mn^2+^ both accumulate to phytotoxic concentrations in flooded porewater. The mechanisms and consequences of this iron and manganese toxicity are detailed in Section 4.4.2. Hydrogen sulfide produced by sulfate-reducing bacteria compounds this stress by directly inhibiting mitochondrial Complex IV, amplifying the respiratory crisis imposed by oxygen depletion. ACC-deaminase-producing PGPR offer a genotype-independent mitigation strategy by reducing root ethylene load across diverse crop systems, though as Section 3.4 discusses, field performance is considerably less consistent than laboratory results suggest (Glick, 2014).

### 4.4 Impaired nutrient assimilation and ion toxicity

#### 4.4.1 Impaired nitrogen assimilation

Nitrogen assimilation depends on a sequence of energy-intensive steps from nitrate reduction through nitrate reductase and nitrite reductase to amino acid synthesis via the glutamine synthetase-glutamate synthase (GS-GOGAT) pathway. Waterlogging disrupts this at multiple points. SnRK1 directly phosphorylates and inactivates nitrate reductase as part of its ATP-conservation strategy, while denitrification in the anaerobic rhizosphere simultaneously depletes the nitrate supply reaching roots (Baena-Gonzalez et al., 2007). The combined effect is a sharp decline in amino acid biosynthesis, particularly of glutamine and asparagine, constraining protein synthesis and limiting the capacity for adaptive growth responses (Vartapetian and Jackson, 1997). Some compensation occurs through the alanine aminotransferase reaction, which converts pyruvate and glutamate into alanine and alpha-ketoglutarate, maintaining partial amino acid flux while recycling carbon into the TCA cycle (Rocha et al., 2010).

#### 4.4.2 Iron and manganese toxicity

Iron toxicity is among the most common micronutrient disorders of wetland rice, with reported yield losses in farmers' fields typically ranging from 15–30% and sometimes reaching complete crop failure (Becker and Asch, 2005). As Fe^2+^ accumulates in flooded porewater, it enters root cells via OsIRT1 and Natural Resistance-Associated Macrophage Protein (NRAMP) transporters. Inside cells, excess Fe^2+^ drives Fenton reactions generating hydroxyl radicals that trigger lipid peroxidation, protein oxidation, and DNA damage. When antioxidant defenses are overwhelmed, the damage becomes visible as leaf bronzing, a reddish-brown discoloration caused by oxidized polyphenol deposits in the leaf mesophyll. Manganese toxicity follows a similar but distinct path. Elevated Mn^2+^ can disturb the uptake and homeostasis of Ca^2+^, Mg^2+^, Zn^2+^, and Fe, impair photosynthesis, and cause interveinal chlorosis and necrotic spotting (Alejandro et al., 2020).

### 4.5 Stomatal regulation and hormonal signaling

Waterlogging impairs root hydraulic conductance through two simultaneous mechanisms. Cytoplasmic acidification causes dephosphorylation of PIP aquaporins, reducing water channel permeability, while falling ATP levels limit active transport processes that sustain osmotic water uptake. The result is a physiological paradox: plants show water stress symptoms including reduced guard cell turgor and partial stomatal closure despite being surrounded by excess water. As described in Section 4.2, xylem-borne and locally regulated leaf ABA activate the PYR/PYL–PP2C–SnRK2–SLAC1 cascade, suppressing photosynthesis at precisely the moment when flooded roots most need photosynthate to sustain fermentative glycolysis (Cutler et al., 2010). Ethylene adds further complexity: trapped in waterlogged tissues, it can promote stomatal closure by stimulating ABA biosynthesis, but in some species it paradoxically drives stomatal opening through EIN3/EIL1-mediated transcriptional programs, with the outcome depending on hormone concentrations and the species-specific signaling architecture (Voesenek and Bailey-Serres, 2015).

## 5 Biochemical adjustments

### 5.1 Ethanolic fermentation and NAD^+^ regeneration

Ethanolic fermentation is the primary survival mechanism for most higher plants during short-term flooding. Its central function is regenerating NAD^*+*^, which is essential for sustaining glycolysis under low oxygen. Alcohol dehydrogenase (ADH) achieves this in the final step by converting acetaldehyde to ethanol, while the upstream enzyme pyruvate decarboxylase (PDC) converts pyruvate to acetaldehyde in a thiamine pyrophosphate-dependent reaction. PDC activity is self-regulating, as rising pyruvate concentrations under low oxygen directly stimulate flux through the pathway, matching output to glycolytic demand (Tadege et al., 1999). Without NAD^*+*^ recycling, the NADH pool becomes fully reduced within minutes and glycolysis halts entirely (Perata and Alpi, 1993).

Lactic fermentation via lactate dehydrogenase operates primarily in the early stages of oxygen deprivation, when cytoplasmic pH is near neutral and enzyme activity is at its peak. As acidification progresses, enzyme kinetics shift in favor of PDC and ADH, naturally limiting further lactate production and preventing worsening acidosis (Roberts et al., 1985). This pH-stat model does not hold uniformly across species, however. Some flood-tolerant aquatic species rely almost entirely on ethanolic fermentation and produce very little lactate. Others sustain high lactate production for extended periods instead. A comparative transcriptomic study across rice, *Arabidopsis*, and poplar found that fermentation gene expression, including lactate dehydrogenase, diverges substantially between species. This divergence does not track cleanly with flooding tolerance (Narsai et al., 2011). The pH-stat model therefore describes a common pattern rather than a universal rule. Additional carbon disposal routes include the alanine aminotransferase reaction, which couples pyruvate with glutamate to produce alanine while sustaining partial TCA cycle flux, and the gamma-aminobutyric acid (GABA) shunt, initiated by Ca^2+^/calmodulin-activated glutamate decarboxylase (Rocha et al., 2010). The metabolic partitioning of pyruvate under hypoxic conditions is illustrated in **Figure 5**.

**Figure 5:**
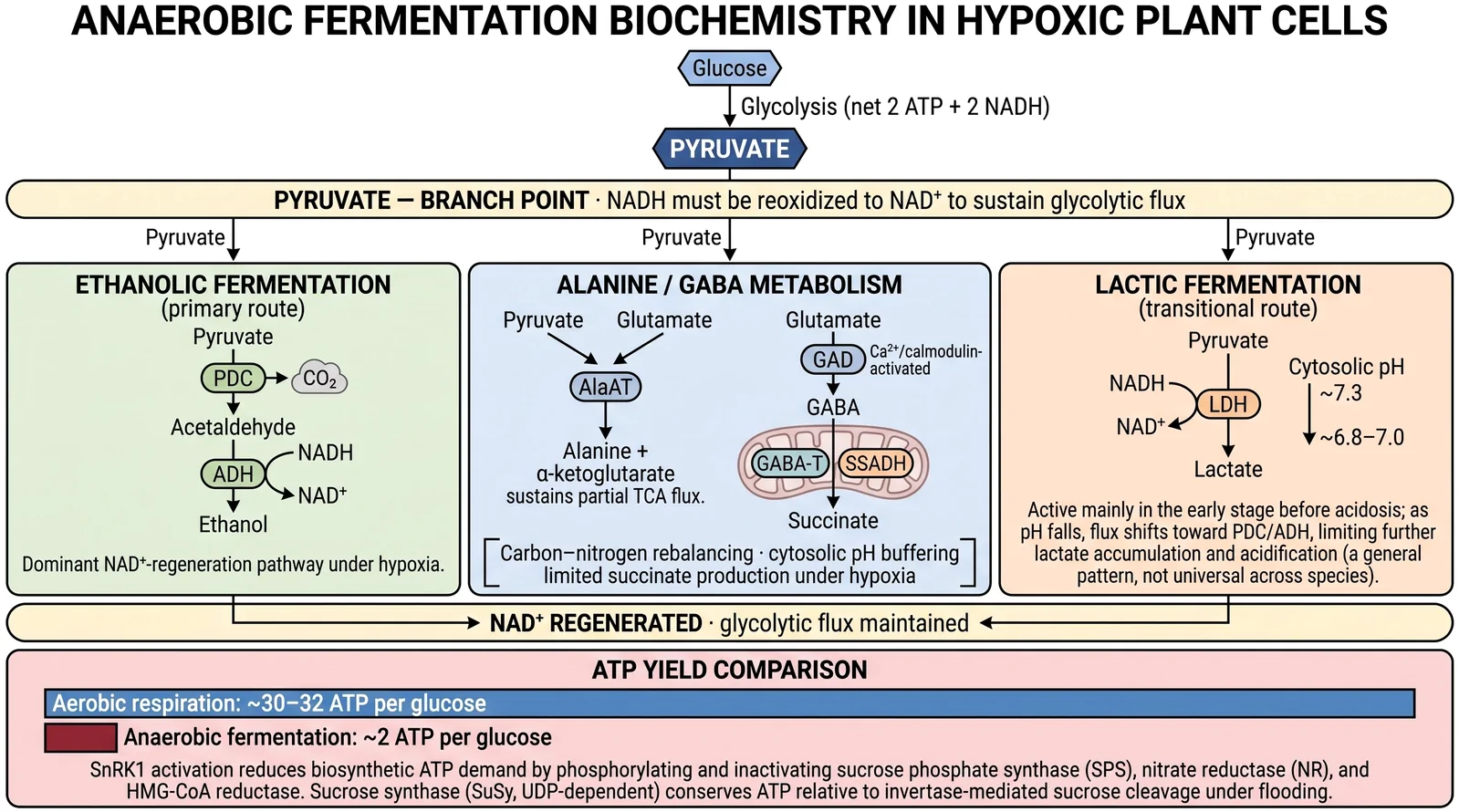
Anaerobic fermentation biochemistry. Pyruvate is partitioned among ethanolic fermentation (PDC → ADH; the principal route for NAD^+^ regeneration; left), lactic fermentation (LDH-mediated NAD⁺ regeneration; transitional and pH-dependent; right), and alanine/GABA metabolism (AlaAT/GAD; carbon–nitrogen rebalancing, cytosolic pH buffering, and limited succinate production; center). Net ATP yield = 2 mol/glucose versus 30–32 aerobic, necessitating SnRK1-mediated metabolic depression. Sucrose synthase (SuSy; UDP-dependent pathway) provides more efficient ATP yield than invertase under flooding. Based on Perata and Alpi (1993); Roberts et al. (1985); Rocha et al. (2010).

In maize and rice, the induction of sucrose synthase under flooding provides a notable metabolic advantage. Sucrose synthase cleaves sucrose via a UDP-dependent reaction that is energetically more efficient than invertase-mediated hydrolysis, improving the net ATP yield from sucrose fermentation (Gibbs and Greenway, 2003). The preferential expression of *PDC* and *ADH* genes in root tips reflects a clear metabolic priority: meristematic cells at the root apex cannot afford to stop dividing during early flooding, and anaerobic gene induction occurs earlier in root tips than in basal root regions across a range of plant species (Dolferus et al., 2011).

### 5.2 Reactive oxygen species and reoxygenation injury

Paradoxically, the most severe oxidative damage in flooded plants often occurs during the reoxygenation phase after the flood recedes rather than during oxygen deprivation itself. This pattern resembles ischemia-reperfusion injury seen in mammalian tissues. When oxygen is reintroduced to tissues with over-reduced mitochondrial electron transport chain components, electrons are transferred to O_2_ at rates exceeding antioxidant capacity, leading to the production of superoxide anion radicals at Complexes I and III, hydrogen peroxide through spontaneous or superoxide dismutase (SOD)-catalyzed reactions, and hydroxyl radicals through Fenton reactions involving accumulated Fe^2+^ (Blokhina et al., 2003). Hydroxyl radicals rapidly react with membrane polyunsaturated fatty acids, triggering chain-reaction lipid peroxidation. Malondialdehyde and 4-hydroxynonenal are widely used as biomarkers of oxidative membrane damage. The generation of reactive oxygen species (ROS) and the antioxidant defense network are depicted in **Figure 6**.

**Figure 6:**
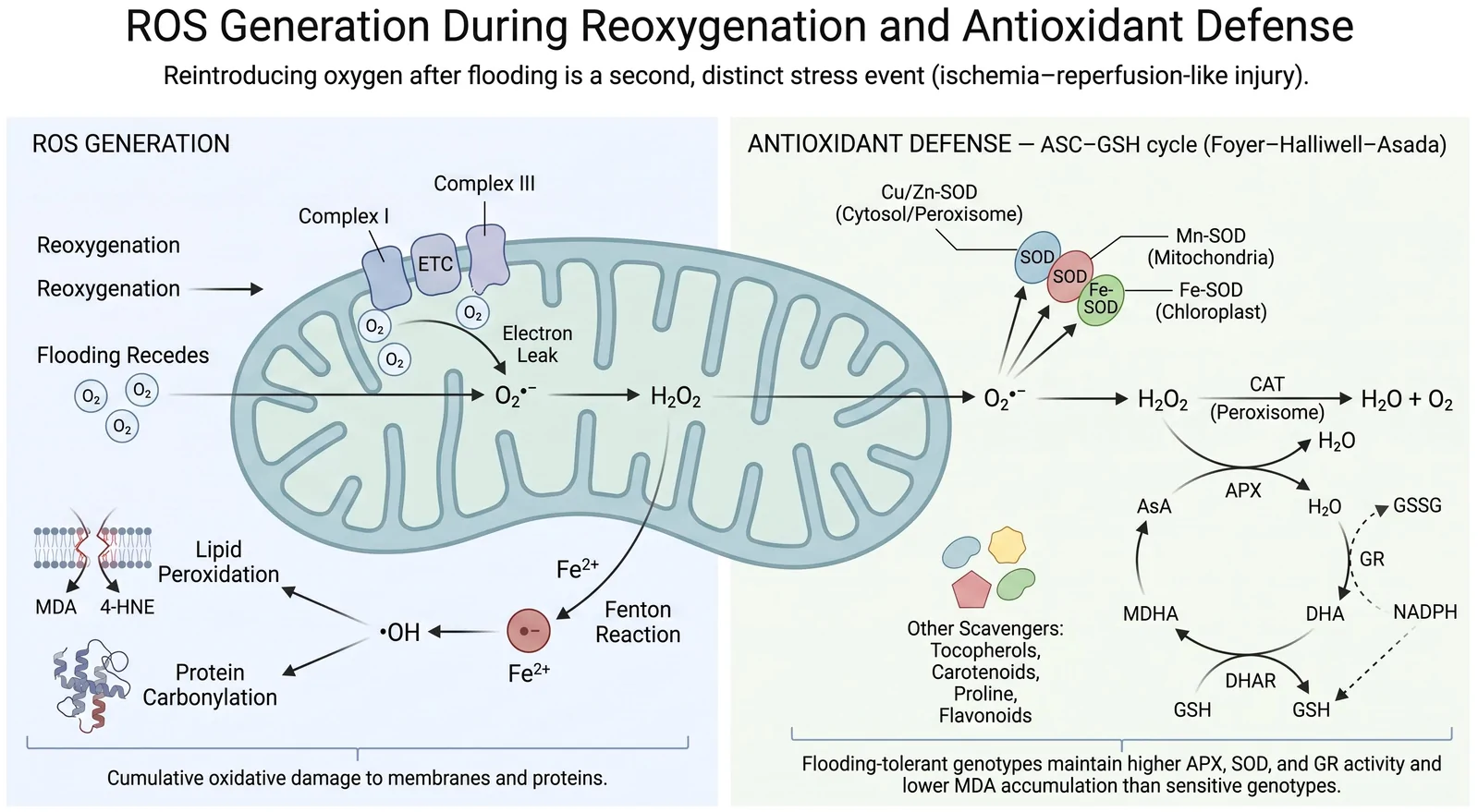
Reactive oxygen species (ROS) dynamics during reoxygenation (left) and antioxidant defense network (right). O_2_ reintroduction to over-reduced electron transport chain (ETC) generates superoxide (O_2_•^−^) at Complexes I and III; Fenton reactions with accumulated Fe^2+^ produce hydroxyl radicals (•OH) causing lipid peroxidation (MDA, 4-HNE) and protein carbonylation. Right: sequential SOD → CAT/APX → Foyer–Halliwell–Asada ASC–GSH cycle detoxification supported by non-enzymatic scavengers (tocopherols, carotenoids, GSH). Tolerant genotypes maintain higher APX, SOD, and GR activity. Based on Mittler (2002); Asada (1999); Blokhina et al. (2003).

### 5.3 Enzymatic and non-enzymatic antioxidant defenses

Enzymatic antioxidant defenses are structured into three sequential levels. Superoxide dismutase isoforms found in the cytosol and peroxisomes (Cu/Zn-SOD), mitochondria (Mn-SOD), and chloroplasts (Fe-SOD) convert O^·*2−*^ to H_2_O_2_ and O_2_, forming the primary enzymatic line of defense against ROS overproduction (Mittler, 2002). Catalase (CAT) in peroxisomes and ascorbate peroxidase (APX), which operates within the Foyer-Halliwell-Asada ascorbate-glutathione cycle, both detoxify H_2_O_2_. Ascorbate peroxidase reduces H_2_O_2_ using ascorbate as an electron donor, the oxidized ascorbate is recycled by monodehydroascorbate reductase (MDHAR) and dehydroascorbate reductase (DHAR) enzymes, and glutathione is regenerated by glutathione reductase (GR) using NADPH, completing the cycle (Asada, 1999).

Flooding-tolerant genotypes consistently maintain higher levels of ascorbate peroxidase, superoxide dismutase, and glutathione reductase activity and mount quicker, stronger responses in these enzymes during both flooding and reoxygenation compared to sensitive genotypes. This correlates with better membrane integrity, lower malondialdehyde accumulation, and faster canopy recovery after flooding (Biemelt et al., 1998). Breeding strategies that enhance antioxidant capacity during the reoxygenation phase by introducing ascorbate peroxidase and glutathione reductase alleles from tolerant sources offer a promising complement to improving survival during submergence itself (Yeung et al., 2018). This pattern extends to legumes as well: waterlogging-responsive expression of antioxidant defense genes has been directly documented in mung bean roots, reinforcing the same tolerant-genotype pattern outside the cereals (Sairam et al., 2011).

### 5.4 Osmolyte accumulation and metabolic reprogramming

#### 5.4.1 Proline accumulation

Proline accumulation under waterlogging serves several protective functions beyond its well-known role in osmotic adjustment. Its synthesis runs primarily through the glutamate pathway, where Δ1-pyrroline-5-carboxylate synthase (P5CS) catalyzes the rate-limiting step, followed by Δ1-pyrroline-5-carboxylate reductase (P5CR) to yield proline. Both enzyme activity and gene expression are induced by flooding-associated signals including elevated cytoplasmic Ca^2+^ and ABA-mediated transcription (Voesenek and Bailey-Serres, 2015). Beyond osmotic balance, proline contributes in three additional ways. Its ring structure enables it to quench singlet oxygen and hydroxyl radicals generated during post-flood reoxygenation. It also acts as a molecular chaperone, stabilizing protein structure and preventing aggregation under stress. During recovery, mitochondrial oxidation of proline back to glutamate donates electrons to the electron transport chain, providing a short-term energy buffer as aerobic metabolism is restored. Flooding-tolerant genotypes of wheat, maize, and soybean consistently accumulate proline faster than sensitive genotypes, correlating with better membrane integrity and reduced electrolyte leakage (Blokhina et al., 2003).

#### 5.4.2 GABA shunt and amino acid rebalancing

GABA accumulates rapidly in hypoxic tissues through glutamate decarboxylase, a Ca^2+^/calmodulin-activated enzyme that converts glutamate to GABA in the cytosol (Rocha et al., 2010). Under waterlogging, the GABA shunt reroutes carbon into the TCA cycle: GABA is transaminated to succinic semialdehyde by GABA transaminase (GABA-T) in the mitochondria, then oxidized to succinate by succinic semialdehyde dehydrogenase (SSADH), re-entering the cycle directly. This sustains partial TCA flux and maintains some mitochondrial membrane potential even when glycolytic carbon is diverted into ethanolic fermentation. The GABA shunt also functions as a pH buffer, as glutamate decarboxylase consumes one proton per reaction, providing biochemical resistance to cytoplasmic acidification when cytosolic pH falls from around 7.3 to 6.8–7.0. Taken together, the interconversion of glutamate, alanine, and GABA forms an integrated stress-response system that simultaneously manages carbon flux, nitrogen recycling, pH homeostasis, and energy balance (Rocha et al., 2010).

#### 5.4.3 Glycine betaine and membrane protection

Glycine betaine, a zwitterionic compound synthesized from choline in chloroplasts, has more limited biosynthesis in rice and several other crops than in halophytes and certain monocot cereals (Voesenek and Bailey-Serres, 2015). Where it does accumulate, it protects flooded leaves in two main ways: stabilizing photosystem II against photo-inhibitory damage, and maintaining membrane fluidity against osmotic stress and Fe^2+^/Mn^2+^-induced lipid peroxidation. In tolerant genotypes, this accumulation correlates with better-preserved photosynthetic quantum yield and lower malondialdehyde levels (Blokhina et al., 2003). **Table 2**, at the end of this section, summarizes glycine betaine alongside the other osmolytes discussed above.

**Table 2:** Osmolytes accumulated under waterlogging: synthesis, protective functions, and crop-specific notes.

Osmolyte	Synthesis/source	Protective function(s)	Notes
Proline	Glutamate pathway via P5CS (rate-limiting) and P5CR; induced by cytoplasmic Ca^2+^ and ABA signaling	Osmotic adjustment; quenches singlet oxygen and hydroxyl radicals; molecular chaperone stabilizing protein structure; mitochondrial re-oxidation to glutamate buffers energy supply during recovery	Tolerant wheat, maize, and soybean genotypes accumulate it faster than sensitive genotypes, correlating with better membrane integrity (Blokhina et al., 2003)
GABA	Glutamate decarboxylase (Ca^2+^/calmodulin-activated), cytosolic	GABA shunt (GABA-T then SSADH) sustains partial TCA flux and mitochondrial membrane potential; buffers cytoplasmic pH by consuming one proton per reaction; supports nitrogen recycling	Part of an integrated glutamate-alanine-GABA system that jointly manages carbon flux, nitrogen recycling, pH, and energy balance (Rocha et al., 2010)
Glycine betaine	Synthesized from choline in chloroplasts	Stabilizes photosystem II against photo-inhibitory damage; maintains membrane fluidity against osmotic stress and Fe^2+^/Mn^2+^-induced lipid peroxidation	Biosynthesis is limited in rice and several other crops relative to halophytes and certain monocot cereals; accumulation correlates with preserved photosynthetic quantum yield (Voesenek and Bailey-Serres, 2015; Blokhina et al., 2003)

#### 5.4.4 SnRK1-mediated metabolic reprogramming

SnRK1 is detailed in Section 3.3.4. It is the plant cell’s central energy sensor. Under waterlogging, it phosphorylates and inactivates key biosynthetic enzymes. This cuts anabolic ATP demand. In this osmolyte-accumulation context, the carbon and nitrogen freed by this shutdown are redirected toward osmolyte synthesis and fermentative ATP generation. **Figure 7** provides an integrated overview of osmolyte accumulation, nutrient imbalance, and stomatal regulation under waterlogging. More broadly, this reprogramming shifts the cell’s priorities away from growth and toward survival through anaerobic glycolysis, fermentation, osmolyte production, and antioxidant defense. SnRK1 effectively acts as a rheostat, calibrating the balance between growth and survival according to the severity and duration of oxygen deprivation (Gibbs and Greenway, 2003). Critically, the entire shift is reversible. When oxygen returns, sucrose delivery resumes, T6P levels recover, SnRK1 activity returns to baseline, and anabolic pathways are progressively restored, allowing the plant to transition back into growth and support yield recovery.

**Figure 7:**
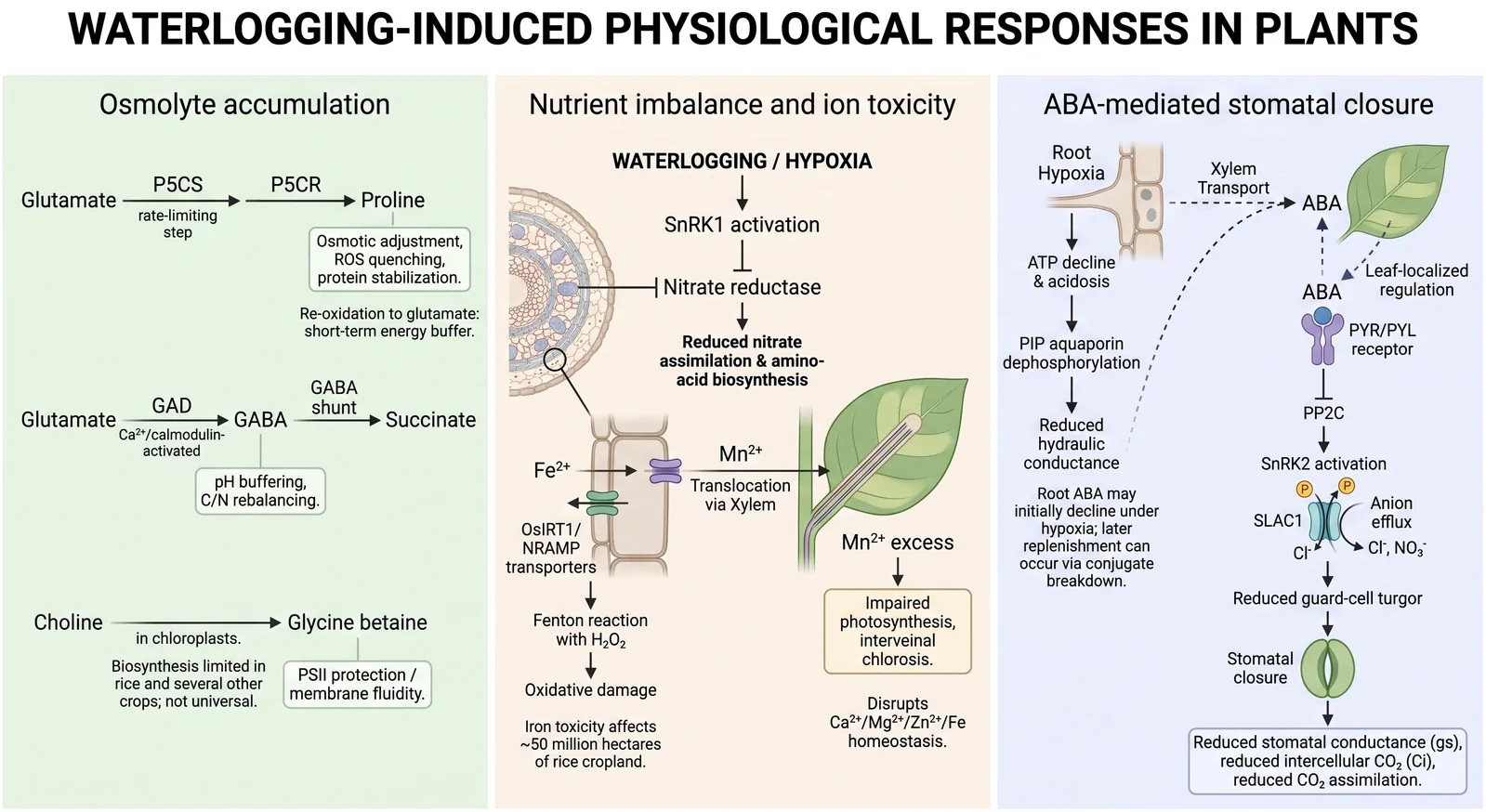
Osmotic and ionic stress responses under waterlogging, in three parts. Left: osmolyte accumulation. Proline, made via P5CS/P5CR, gives osmotic protection, quenches ROS, and acts as a chaperone. GABA, produced through calcium-activated GAD, helps rebalance carbon and nitrogen and buffers pH. Glycine betaine contributes to membrane and photosystem II protection. Center: nutrient imbalance. Nitrogen assimilation is impaired through SnRK1-mediated inhibition of nitrate reductase, iron toxicity arises from Fenton reactions, and manganese toxicity disrupts Ca^2+^, Mg^2+^, Zn^2+^, and Fe homeostasis, thereby impairing photosynthesis and contributing to chlorosis. Right: ABA-mediated stomatal closure, involving aquaporin dephosphorylation and the PYR/PYL–PP2C–SnRK2–SLAC1 cascade that reduces guard cell turgor. Based on Rocha et al. (2010); Felle (2005); Cutler et al. (2010).

**Table 2** summarizes and compares the three osmolytes discussed above, drawing together their synthesis routes, protective functions, and crop-specific notes in one place.

## 6 Morphological and anatomical adaptations

### 6.1 Aerenchyma: function, formation, and ecological significance

#### 6.1.1 Lysigenous aerenchyma via programmed cell death

Lysigenous aerenchyma is the main type found in maize, wheat, barley, soybean, and *Arabidopsis*. It forms through the selective death of cortical cells in submerged roots. In maize, cortical gas space expands from 2–3% in well-aerated roots to 20–30% within 48–96 h of flooding. The exact magnitude and timing vary with genotype, plant age, and conditions such as temperature and soil oxygen status, so these figures are representative rather than universal. This expansion is driven by ethylene-triggered calcium influx and hydrogen peroxide production in cells destined for programmed cell death. The death process involves metacaspase activation, tonoplast rupture mediated by vacuolar processing enzymes, cytoplasmic acidification, and complete breakdown of cell contents, leaving gas-filled spaces surrounded by intact cell layers (Drew et al., 2000). Ethylene is a central regulator of lysigenous aerenchyma formation under waterlogging (Yamauchi et al. 2018). The pathways of lysigenous and schizogenous aerenchyma formation are illustrated in **Figure 8**.

**Figure 8:**
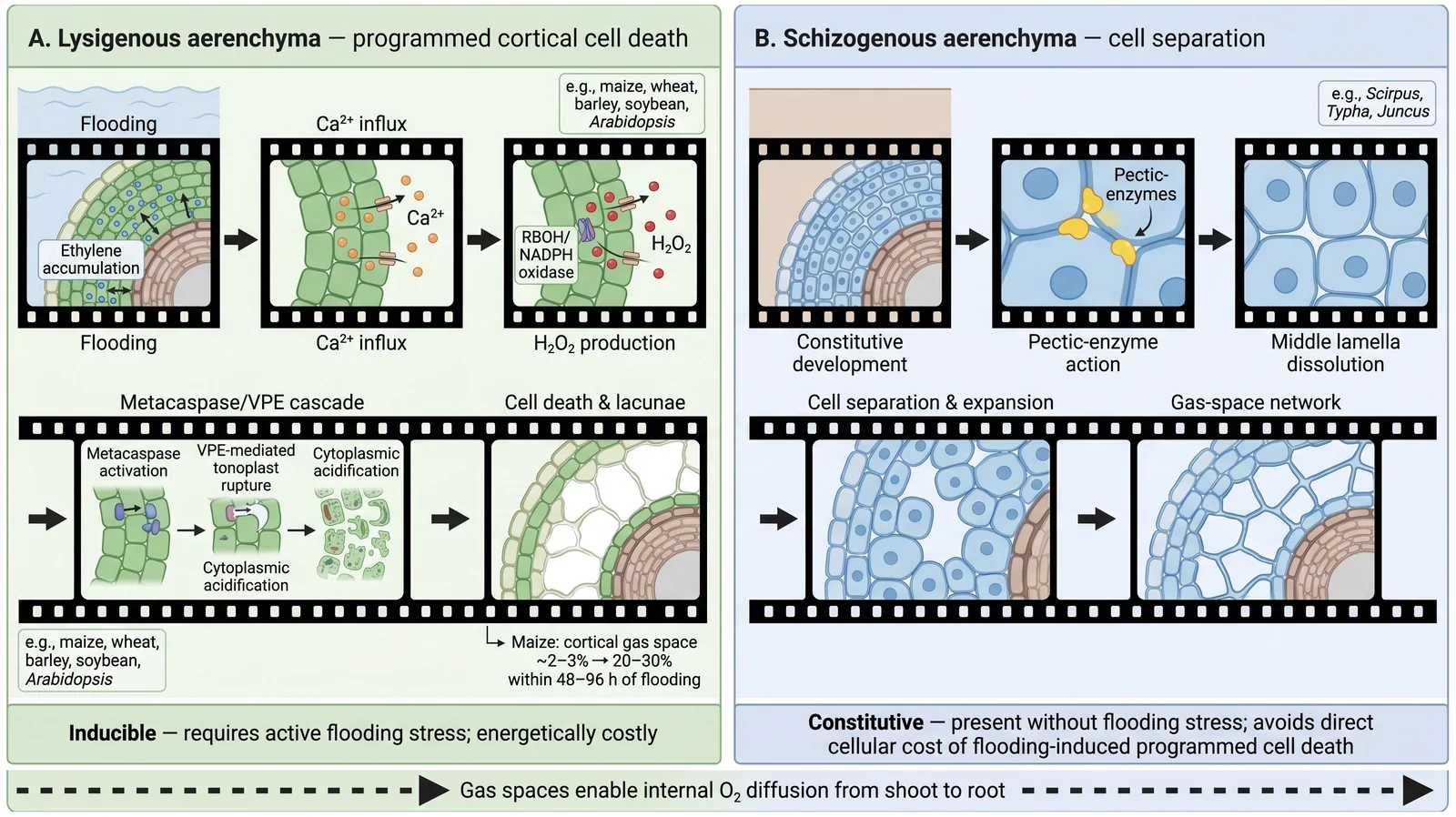
Aerenchyma formation pathways. **(A)** Lysigenous (left): ethylene → Ca^2+^ influx → RBOH/NADPH-oxidase → H_2_O_2_ → vacuolar processing enzyme (VPE) → tonoplast rupture → cell autolysis cascade; in maize, cortical porosity can increase from approximately 3% to 20–30% within 48–96 h of flooding (values vary with genotype and growth conditions). **(B)** Schizogenous (right): constitutive pectic-enzyme-mediated cell separation without programmed cell death, characteristic of obligate wetland taxa including *Scirpus* (a sedge), *Typha* (cattail), and *Juncus* (rush); avoids the direct cellular cost of flooding-induced programmed cell death and can provide gas channels early in development. Based on Drew et al. (2000); Justin and Armstrong (1987); Yamauchi et al. (2018).

#### 6.1.2 Schizogenous aerenchyma via cell separation

Schizogenous aerenchyma is found in obligate wetland plants such as Scirpus (sedge), Typha (cattail), and Juncus (rush). It forms through pectic enzyme activity that dissolves cell-to-cell adhesion, followed by cell expansion forces that create intercellular spaces without killing the cells (Justin and Armstrong, 1987). This process is largely constitutive in obligate wetland species and does not require flooding stress for its initiation, reflecting their evolutionary adaptation to persistently saturated conditions. Because no cell death is involved, schizogenous aerenchyma is less energetically costly per unit of gas space than lysigenous aerenchyma. It also lacks the developmental delays associated with programmed cell death, providing functional gas channels from seedling emergence onward (Colmer and Voesenek, 2009).

#### 6.1.3 Barrier to radial oxygen loss

Aerenchyma alone cannot sustain root-tip oxygenation in anoxic sediments. As oxygen diffuses from shoot to root tip through the aerenchyma, it can leak radially outward across the root cortex before it gets there, especially from older, more permeable root zones. Many wetland and flood-tolerant species counter this with an inducible barrier to radial oxygen loss (ROL): a suberized, often lignified layer that develops in the outer cortex or exodermis under waterlogging. This barrier forces oxygen to travel further down the aerenchyma core instead of escaping locally, preserving enough for aerobic respiration at the growing root tip (Colmer, 2003). The capacity to form a tight ROL barrier is one of the clearest differences between tolerant and sensitive species. Rice, deepwater rice, and many wetland grasses form a strong, inducible barrier. Most upland cereals form little or no barrier and lose oxygen along most of the root length instead, a basal weakness in root aeration recognized in early work on plant gas transport (Armstrong, 1980). Because the barrier also restricts inward diffusion, it limits how much Fe^2+^ and other rhizosphere phytotoxins can reach the root, giving it a second, protective role alongside oxygen conservation.

### 6.2 Adventitious root growth and stem lenticel hypertrophy

Adventitious roots arising from stem nodes above the anaerobic soil layer provide flooded plants with a functional root system that bypasses the oxygen-depleted primary root zone. These roots develop with a high density of aerenchyma, rapidly restoring water and nutrient transport connections disrupted by primary root damage (Vartapetian and Jackson, 1997). Adventitious root development is initiated when ethylene accumulates in the stem, triggering YUCCA family flavin monooxygenases in cortical tissue, increasing indole-3-acetic acid (IAA) production while reducing auxin transport protein activity, leading to IAA accumulation at the waterline (Vidoz et al., 2010). Auxin promotes root primordia formation by degrading repressor proteins targeting Auxin Response Factor (ARF) genes, while gibberellins support primordia emergence by promoting cell wall loosening and DELLA protein degradation at the stem surface (Davière and Achard, 2013).

Lenticel hypertrophy driven by ethylene at the waterline increases gas exchange capacity by replacing tightly arranged cells with loosely connected, highly porous parenchyma cells. These enlarged lenticels serve as entry points for gases into the stem aerenchyma network, completing the gaseous pathway from the atmosphere to submerged roots and enabling aerobic ATP production in root cells even when surrounding soil is fully anoxic (Voesenek and Bailey-Serres, 2015).

### 6.3 Escape versus quiescence: contrasting survival strategies

Two main strategies govern shoot responses to complete submergence. The escape strategy involves rapid internode elongation to reach the water surface for gas exchange, whereas the quiescence strategy restrains growth to conserve carbohydrate reserves for post-flood recovery (Voesenek and Bailey-Serres, 2015). The escape strategy, used by deepwater rice varieties and *Rumex palustris*, depends on ethylene-mediated activation of *the SNORKEL genes SK1 and SK2*, which increase expression of GA20ox2 and promote accumulation of bioactive GA, predominantly GA₁. These gibberellins promote DELLA-protein degradation, releasing EXPANSIN activity and driving internode elongation to keep the shoot tip above rising water (Hattori et al., 2009). In contrast, the quiescence strategy used by Sub1A-1 rice varieties reduces GA responsiveness through DELLA-mediated growth repression, including the involvement of SLR1 and SLRL1, thereby restricting EXPANSIN-associated elongation. This response helps conserve starch and soluble-sugar reserves, supporting survival during transient complete submergence and vigorous recovery after floodwater recedes (Fukao et al., 2006). These contrasting strategies are adaptive in different flooding environments: escape is advantageous under rising-water conditions in which shoots can reach the water surface, whereas quiescence is primarily advantageous during transient complete submergence. These contrasting strategies are compared in **Figure 9**.

**Figure 9:**
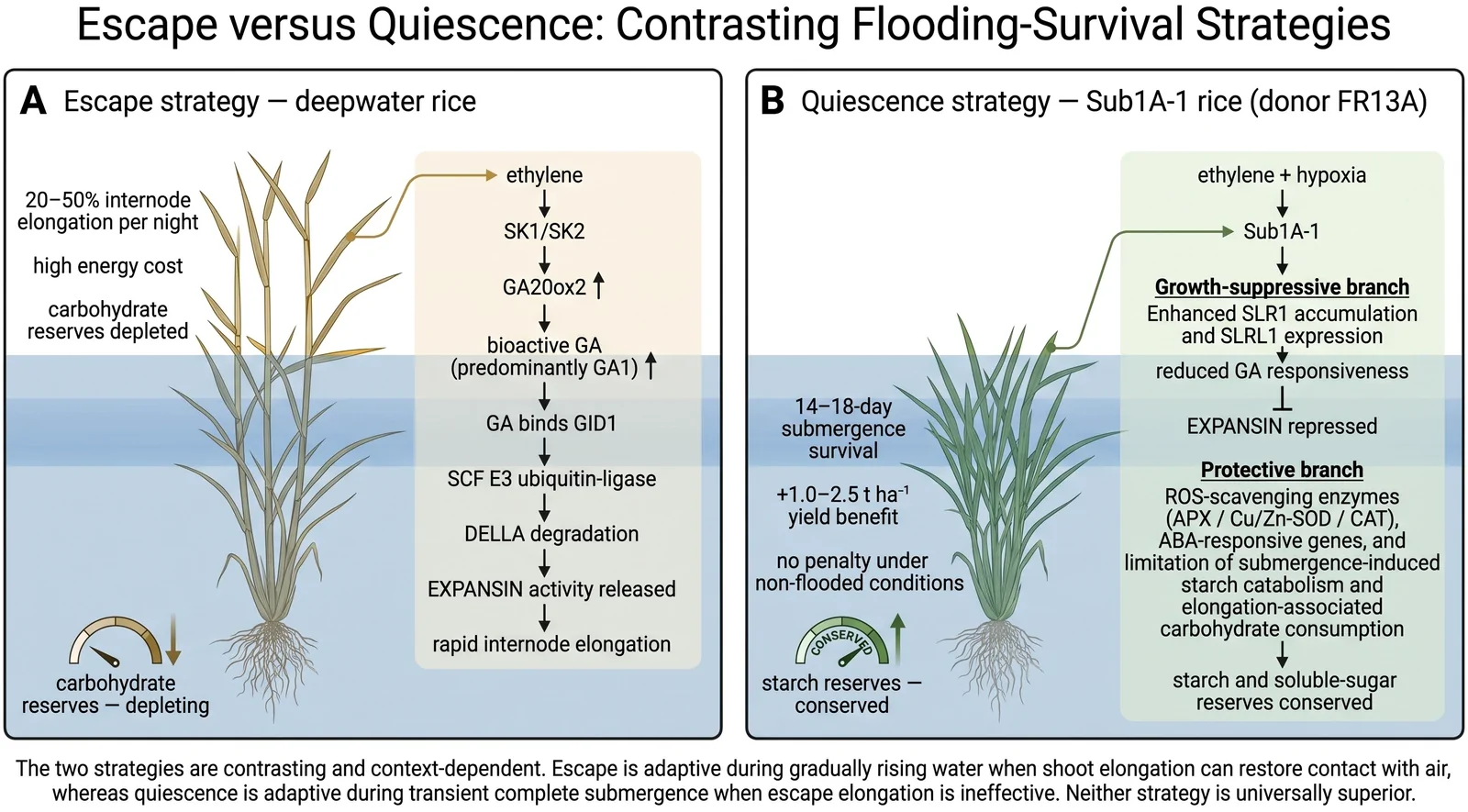
Escape versus quiescence strategies. **(A)** Escape (left): ethylene activates *SK1/SK2* and *GA20ox2*, promoting accumulation of bioactive GA, predominantly GA₁, DELLA-protein degradation, and EXPANSIN activation. This drives internode elongation at high energy cost and can deplete carbohydrate reserves. **(B)** Quiescence (right): Sub1A-1 promotes DELLA-mediated suppression of GA responsiveness, including the involvement of SLR1 and SLRL1; EXPANSIN activity is restrained; antioxidant enzymes (APX/SOD/CAT) are upregulated; and carbohydrate reserves are conserved. Both strategies represent distinct flooding adaptations: escape is advantageous where shoots can outgrow rising water, whereas quiescence conserves resources during transient complete submergence (van Veen et al., 2013; Ismail et al., 2013).

## 7 Hormonal regulation under hypoxia

### 7.1 Ethylene as central flooding signal

Ethylene plays a central role in the hormonal response to flooding. Its accumulation in submerged tissues occurs automatically, scaling with flood depth and duration, and is perceived through signaling pathways involving Ethylene Response (ETR) receptors, EIN2, and EIN3/EIL1 transcription factors (Voesenek and Bailey-Serres, 2015). Flooding triggers changes in cytosolic calcium that activate calcium-dependent protein kinases (CDPKs), which phosphorylate and stabilize ACC synthase proteins, accelerating the production of ACC from S-adenosylmethionine. Because ACC oxidase requires molecular oxygen to function and is inactive in oxygen-deprived root tissues, ACC produced in the roots is transported through the xylem to shoot tissues where ACC oxidase converts it to ethylene. This spatial separation of ACC production in flooded roots and ethylene generation in oxygen-rich shoots creates a signaling circuit that coordinates the whole-plant flooding response despite anaerobic conditions in the roots (Hartman et al., 2021). The complex hormonal interaction network governing flooding responses is shown in **Figure 10**.

**Figure 10:**
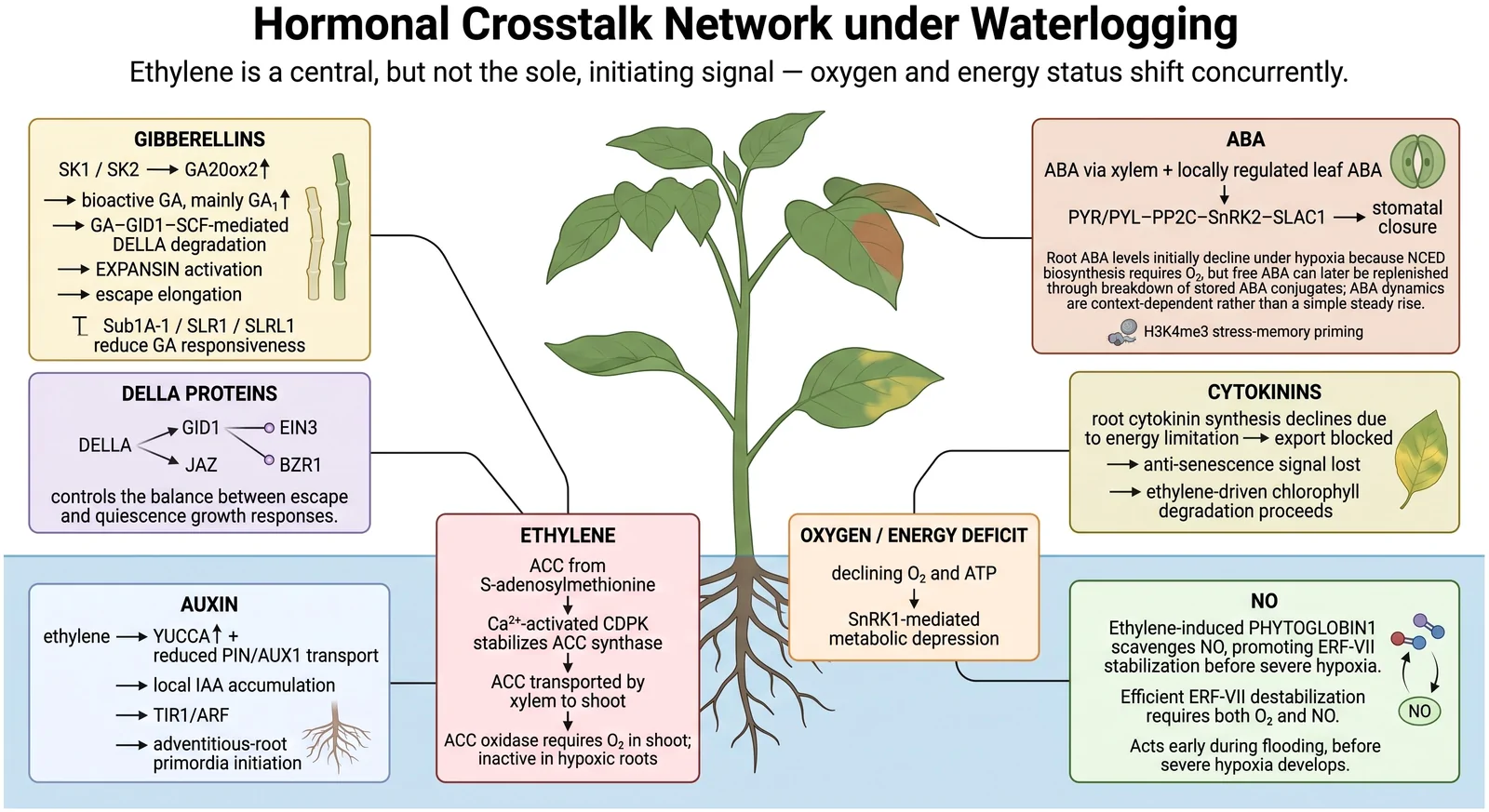
Hormonal crosstalk network. Ethylene (center) is a primary flooding signal and interacts bidirectionally with auxin (adventitious-root induction via the YUCCA/IAA/ARF pathway), gibberellins (escape/quiescence regulation through DELLA-dependent growth control), ABA (stomatal closure via the PYR/PYL–PP2C–SnRK2–SLAC1 cascade; stress-memory priming via H3K4me3), and cytokinins (loss of anti-senescence signaling promotes chlorophyll degradation). DELLA proteins integrate GA, EIN3, JAZ, and BZR1 inputs. Efficient ERF-VII degradation requires both O_2_ and NO; reduced availability of either gas can stabilize ERF-VII by limiting N-degron-mediated proteolysis. Based on Voesenek and Bailey-Serres (2015); Davière and Achard (2013); Marín-de la Rosa et al. (2014); and Lämke and Bäurle (2017).

### 7.2 Gibberellin-auxin crosstalk in developmental responses

The interactions among gibberellins, auxin, and ethylene are most evident in adventitious root formation and the escape elongation response. As introduced in Section 6.2, ethylene promotes IAA accumulation at the waterline by stimulating YUCCA activity and reducing PIN/AUX1 transport. IAA specifies root primordia identity through TIR1/ARF pathways, while gibberellins promote primordia emergence by inducing epidermal cell death and degrading DELLA proteins overlying the primordium (Vidoz et al., 2010; Steffens and Sauter, 2005). In the escape elongation response, ethylene activates SNORKEL proteins SK1 and SK2, which increase expression of *GA20ox2*, and promote accumulation of bioactive GA, predominantly GA_1_. These gibberellins facilitate DELLA-repressor degradation and trigger cell elongation through EXPANSIN activation (Hattori et al., 2009). DELLA proteins act as integrators of multiple hormonal signals, interacting not only with GID1, the GA receptor, but also with EIN3, JAZ, and BZR1, positioning them as key regulators that balance growth responses according to diverse hormonal inputs (Davière and Achard, 2013).

Phylogenomic studies have shown that the interaction between ERF and DELLA proteins is an evolutionarily conserved mechanism found in both monocots and dicots, supporting their role as fundamental regulators of growth during stress across flowering plant history (Marín-de la Rosa et al., 2014). This conservation suggests that modifying DELLA levels through natural variation or targeted breeding could offer a practical approach to adjusting the balance between escape and quiescence strategies in flood-prone crop species beyond rice.

### 7.3 ABA, cytokinins, and epigenetic stress memory

ABA plays context-dependent roles in flooding stress. Initially, ABA levels in roots decline under flooding because the key biosynthetic enzyme 9-cis-epoxycarotenoid dioxygenase (NCED) requires molecular oxygen, which is limited under waterlogged conditions. However, breakdown of stored ABA conjugates can replenish free ABA during extended oxygen deficiency (Fukao and Bailey-Serres, 2008). At moderate concentrations, ABA promotes stomatal closure and interacts with the Sub1A quiescence program by activating ROS-protective genes. Concurrently, cytokinin production in roots declines substantially during waterlogging due to energy limitations, removing anti-senescence signals from leaves and allowing ethylene-driven chlorophyll breakdown to proceed unchecked (Vartapetian and Jackson, 1997).

Epigenetic stress memory refers to enhanced performance during a second flooding event following prior flood exposure. This improved response may involve ABA-dependent deposition of chromatin marks such as H3K4me3, a priming mechanism well documented for other abiotic stresses including heat, salt, and desiccation (Lämke and Bäurle, 2017), though direct evidence for the same marks at flooding-response genes specifically remains limited. This stress memory has practical relevance in regions where crops face repeated flooding within a single growing season. The reoxygenation phase following flooding is increasingly recognized as a distinct stress period requiring its own regulatory attention, as oxidative damage patterns during recovery differ from those imposed during submergence itself (Yeung et al., 2018).

## 8 Genetic and genomic basis of tolerance

### 8.1 QTL landscape across major crop species

Flooding tolerance is a complex genetic trait influenced by multiple quantitative trait loci (QTL) that interact depending on genetic background, developmental stage, flooding conditions, and post-flooding recovery environment (Setter and Waters, 2003). In rice, the Sub1 QTL on chromosome 9 is the most well-characterized flooding tolerance locus in any crop. It contains closely linked *Sub1A*, *Sub1B*, and *Sub1C* ERF-VII genes, with the Sub1A-1 allele from the tolerant donor FR13A being essential for submergence tolerance (Xu and Mackill, 1996). For anaerobic germination, fine mapping pinpointed the AG1.1 QTL on chromosome 9 to *OsTPP7*, a trehalose-6-phosphate phosphatase gene involved in starch mobilization under anaerobic conditions (Kretzschmar et al., 2015). The genetic loci and primary mechanisms associated with flooding tolerance are summarized in **Table 3**.

**Table 3:** Genetic loci and primary mechanisms of waterlogging/flooding tolerance in major crop species.

Crop	Key gene/QTL	Primary tolerance mechanism	Key reference
Rice (Oryza sativa)	*Sub1A-1 (chr. 9); SK1/SK2; OsTPP7*	Submergence quiescence; deepwater escape; anaerobic germination	Xu and Mackill (1996); Hattori et al. (2009); Kretzschmar et al. (2015)
Wheat (T. aestivum)	*QTLs on chr. 4A, 6D, 7B; TaADH1*	Aerenchyma induction; fermentation capacity; root porosity	Setter and Waters (2003)
Maize (Zea mays)	*Aerenchyma QTLs chr. 1 & 5; ZmADH*	Rapid cortical aerenchyma; ADH/PDC induction	Mano and Omori (2007)
Soybean (Glycine max)	Sat_064 QTL (chr. 11)	Adventitious roots; waterlogged nodule protection	Cornelious et al. (2005)
Barley (H. vulgare)	Waterlogging-tolerance QTLs and anaerobic metabolism traits	Fermentative enzyme capacity; aerenchyma induction	Setter and Waters (2003)

QTL, quantitative trait locus; chr., chromosome. References cited within table are primary sources for each crop.

In wheat, QTLs related to waterlogging tolerance and root aerenchyma induction have been identified on chromosomes 4A, 6D, and 7B. Variation in the *TaADH1* gene and other aerenchyma-related loci contributes to differences in root porosity and post-waterlogging yield observed in field studies (Setter and Waters, 2003). For maize, aerenchyma QTLs on chromosomes 1 and 5 explain variation in cortical gas space among inbred lines, and marker-assisted backcrossing of these QTLs into elite strains has improved waterlogging tolerance under field conditions (Mano and Omori, 2007).

### 8.2 Sub1A: mechanism, varieties, and agricultural impact

Sub1A-1 acts as a dual-function transcriptional regulator that activates protective genes while suppressing energy-intensive growth, creating a quiescence state that substantially improves submergence survival. On the protective side, Sub1A-1 increases levels of ROS-scavenging enzymes including ascorbate peroxidase, Cu/Zn-SOD, and catalase, and promotes ABA-responsive gene expression to strengthen antioxidant and quiescence programs. On the growth-suppressive side, Sub1A-1 promotes DELLA-mediated repression of GA responsiveness, including the involvement of SLR1 and SLRL1, and represses *EXPANSIN* genes that would otherwise promote elongation (Fukao et al., 2006). Additionally, Sub1A-1 limits submergence-induced starch catabolism and elongation-associated carbohydrate consumption, helping to conserve starch and soluble-sugar reserves that support rapid regrowth, tillering, and grain filling after floodwater recedes. Post-flood recovery is therefore as important as survival under water (Locke et al., 2018).

Marker-assisted backcross breeding has introduced Sub1A-1 from donor FR13A into popular high-yielding varieties including Swarna, Samba Mahsuri, BR11, IR64, and Thadokkham 1, maintaining over 95% of the recurrent parent genome at non-Sub1 loci while fixing the Sub1 region (Septiningsih et al., 2009). Field trials across India, Bangladesh, Indonesia, and the Philippines showed yield increases of 1.0–2.5 t ha*−1* in Sub1 varieties after 14–18 days of complete submergence, conditions that cause total yield loss in non-Sub1 varieties. Critically, no yield penalty was observed under non-flooded conditions, which is essential for farmers who cannot predict flooding within a growing season (Ismail et al., 2013).

### 8.3 Multi-omics integration and gene regulatory networks

Cell-type-specific translatome profiling and gene-regulatory-network studies have revealed both shared and cell-type-specific hypoxia responses in roots (Mustroph et al., 2009; Reynoso et al., 2019). Gene regulatory network analysis through Assay for Transposase-Accessible Chromatin using sequencing (ATAC-seq) chromatin accessibility and DNA Affinity Purification sequencing (DAP-seq) transcription factor binding data has detailed the regulatory networks controlling cell-type-specific flooding responses at single-gene resolution, revealing a complex web of co-regulators, chromatin remodelers, and transcriptional programs downstream of ERF-VII (Reynoso et al., 2019). Phosphoproteomic studies during hypoxia have demonstrated how SnRK1, CDPK, and Mitogen-Activated Protein (MAP) kinases create extensive post-translational regulatory networks that alter cellular metabolism in ways transcriptomics cannot detect alone (Baena-Gonzalez et al., 2007). Metabolomic profiling using Nuclear Magnetic Resonance (NMR), Gas Chromatography-Mass Spectrometry (GC-MS), and Capillary Electrophoresis-Mass Spectrometry (CE-MS) has measured key metabolic shifts: alanine, GABA, succinate, and ethanol accumulate rapidly after flooding, while TCA cycle intermediates decline, with distinct metabolite signatures helping differentiate tolerant from sensitive genotypes (Rocha et al., 2010). The conservation of this multi-omics response across rice, *Arabidopsis*, poplar, and lotus has been systematically documented (Narsai et al., 2011). These integrated transcriptomic, chromatin-accessibility, phosphoproteomic, and metabolomic layers are summarized in **Figure 11**.

**Figure 11:**
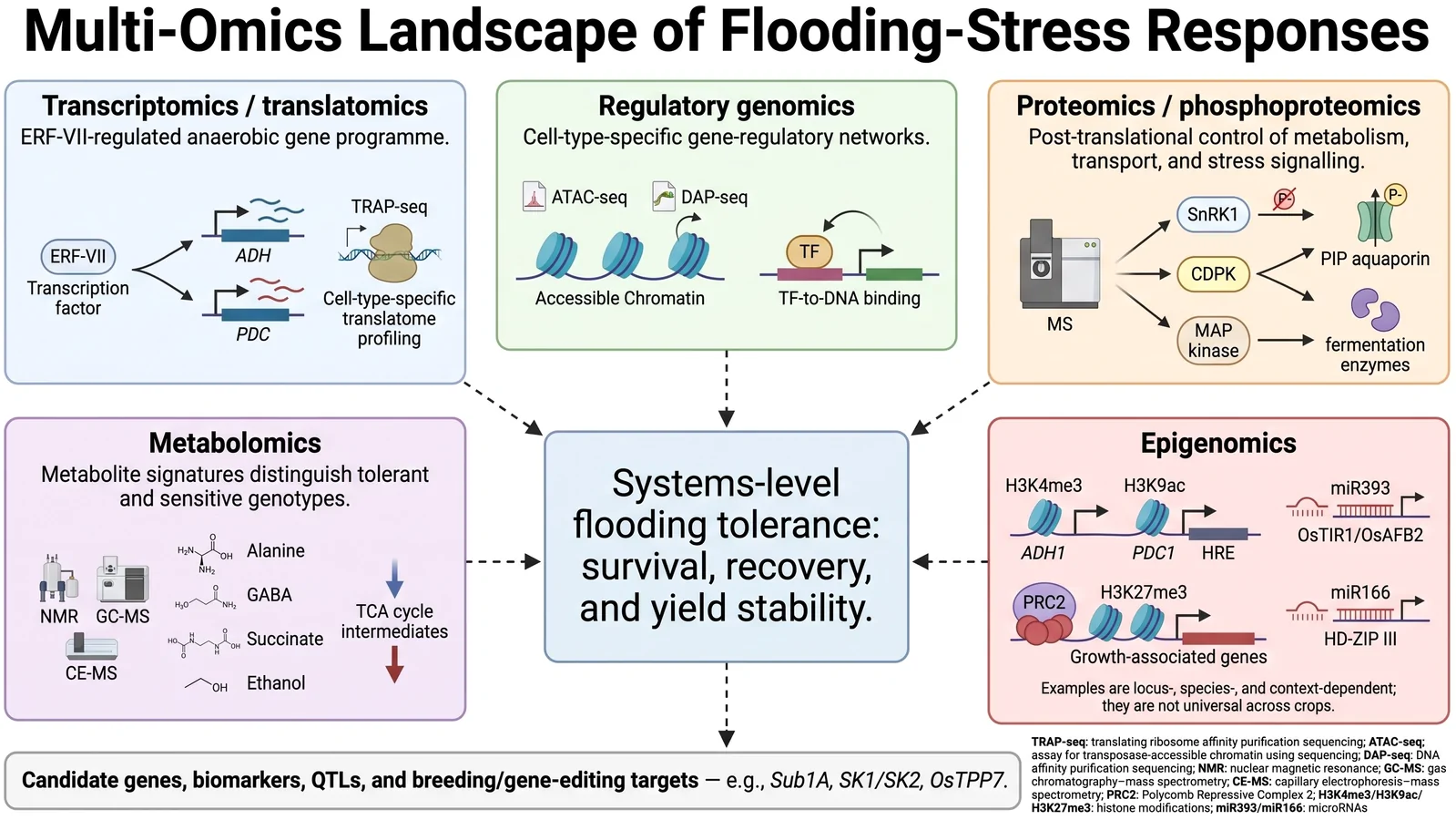
Multi-omics landscape of flooding-stress responses. Five integrated layers include transcriptomics/translatomics (hypoxia-responsive gene regulation; TRAP-seq), regulatory genomics (chromatin accessibility assessed by ATAC-seq and transcription-factor binding assessed by DAP-seq), proteomics/phosphoproteomics (SnRK1/CDPK signaling cascades; fermentation-enzyme regulation; aquaporin dephosphorylation), metabolomics (alanine/GABA/succinate/ethanol accumulation; NMR/GC-MS/CE-MS platforms), and epigenomics (locus- and species-dependent H3K4me3/H3K9ac changes; PRC2/H3K27me3 at growth-associated genes; miR393/miR166). These layers converge on systems-level tolerance. Based on Mustroph et al. (2009); Mustroph et al. (2010); Reynoso et al. (2019); Rocha et al. (2010); and Jiang et al. (2023). H3K4me3/H3K9ac dynamics shown here reflect chromatin-mark mechanisms documented broadly across abiotic stresses, with direct flooding-specific evidence still limited.

### 8.4 Epigenetic regulation and transgenerational flooding memory

Rapid, locus- and species-dependent chromatin changes have been reported during anaerobic gene activation. Permissive H3K4me3 and H3K9 acetylation marks appear at *ADH1*, *PDC1*, and *HRE* loci within hours of flooding, initiated by histone acetyltransferases and methyltransferases recruited by ERF-VII proteins. Conversely, growth-related genes accumulate repressive H3K27me3 marks from Polycomb Repressive Complex 2, leading to stable chromatin silencing during submergence (Lämke and Bäurle, 2017). As introduced in Section 7.3, these chromatin changes underlie the epigenetic stress memory that primes faster and stronger anaerobic responses upon re-flooding. Flood-responsive microRNAs contribute to post-transcriptional regulation. miR393 targets the auxin receptor OsTIR1/OsAFB2, affecting auxin sensitivity and adventitious root development, while miR166 impacts vascular development in flooded tissues by targeting HD-ZIP III transcription factors. A broader small-RNA and mRNA profiling study in eggplant identified 246 differentially expressed microRNAs during waterlogging, with the miR156, miR166, miR167, and miR399 families most strongly affected, indicating that microRNA involvement in the hypoxia response extends well beyond the handful of loci characterized in rice (Jiang et al., 2023). Research into transgenerational epigenetic priming indicates that parental flooding events can enhance offspring flooding responses through inheritable chromatin changes, though this area requires further validation before application in breeding programs (Lämke and Bäurle, 2017).

Combining stacked tolerance alleles in elite rice varieties is a key breeding goal supported by international agricultural programs. This strategy incorporates either Sub1A for submergence tolerance or SK1/SK2 for deepwater escape, alongside OsTPP7 for anaerobic germination and QTLs for reoxygenation tolerance (Bailey-Serres et al., 2019).

### 8.5 Anaerobic germination genes

Anaerobic germination, the capacity of seeds to initiate and complete germination under flooded soil conditions where oxygen availability is severely restricted, is a distinct tolerance trait from submergence tolerance in established plants and is controlled by separate genetic loci. In rice, the primary genetic determinant of anaerobic germination tolerance has been fine-mapped to the AG1.1 QTL on chromosome 9, encoding *OsTPP7*. This enzyme catalyzes the conversion of trehalose-6-phosphate to trehalose, lowering T6P concentrations in germinating seeds under anaerobic conditions (Kretzschmar et al., 2015). By reducing T6P levels, OsTPP7 activates SnRK1, which promotes starch mobilization and enhances glycolytic flux in the germinating embryo, allowing it to generate sufficient substrate-level ATP through fermentative glycolysis to drive cell division, coleoptile elongation, and seedling emergence. In tolerant varieties carrying the functional OsTPP7 allele, germination rate and seedling establishment under flooded direct-seeding conditions are substantially higher, enabling direct-seeded cultivation in flood-prone paddy environments (Kretzschmar et al., 2015). In wheat and maize, homologous *TPP* genes have been identified, but their specific roles in anaerobic germination remain incompletely characterized, representing a significant gap in the genetic knowledge base for non-rice cereal improvement. Barley shows this gap starting to close, though not in the way AG1’s rice biology might predict. A 2025 genome-wide association study mapped post-flooding germination capacity in barley to a locus on chromosome 1, distinct from the chromosome 9 location of rice *AG1*. Rather than trehalose metabolism, the barley locus appears to act through seed coat composition and permeability, which determines whether a seed enters hypoxia-induced secondary dormancy after flooding (Gómez-Álvarez et al., 2025). This suggests that anaerobic germination tolerance may have evolved through more than one genetic route across cereals, and that wheat and barley breeding programs may need their own dedicated gene discovery efforts rather than assuming AG1 orthologs will transfer directly.

### 8.6 Comparative tolerance: hypoxia sensitivity in wheat and maize

#### 8.6.1 Hypoxia sensitivity in *Triticum aestivum* (wheat)

Wheat is among the most waterlogging-sensitive cereal crops, and its sensitivity derives from several interconnected factors that distinguish it mechanistically from tolerant species such as rice. Root cortical porosity in wheat increases from approximately 2–3% to only 10–15% under flooding, compared to 15–30% in rice (Setter and Waters, 2003). This restricted aerenchyma formation results from lower ethylene sensitivity in cortical cells and less robust induction of ethylene biosynthesis genes in response to the anaerobic signal cascade. Consequently, internal oxygen supply to wheat root tips remains inadequate under prolonged flooding, constraining aerobic ATP generation and accelerating tissue death. Wheat also shows limited inducibility of anaerobic fermentation enzymes compared to rice. Although wheat possesses functional ADH encoded by *TaADH1* and PDC genes, their transcriptional induction under hypoxia is slower and reaches lower maximum expression levels, meaning NAD^*+*^ recycling capacity and glycolytic ATP generation are less well sustained under waterlogging. Together, restricted root aeration and limited metabolic acclimation contribute to reduced tillering, premature senescence, and yield loss under waterlogging (Setter and Waters, 2003; Pais et al., 2023). Field-level consequences include reduced tiller production, premature leaf senescence, and yield losses of 25-30% under waterlogging lasting seven or more days.

#### 8.6.2 Responses in *Zea mays* (maize)

Maize occupies an intermediate position on the flooding tolerance spectrum: considerably more sensitive than rice but more tolerant than wheat, largely due to its superior aerenchyma formation capacity. Maize roots rapidly develop extensive lysigenous aerenchyma under waterlogging, with cortical porosity increasing from 3% to 25–30% within 48–96 h (figures that vary by genotype and growth stage, as noted in Section 6.1.1) due to high ethylene sensitivity and robust induction of the Ca^2+^/ROS-mediated programmed cell death cascade in cortical cells (Drew et al., 2000). QTLs controlling aerenchyma formation have been mapped to chromosomes 1 and 5, and marker-assisted transfer of these QTLs into elite inbred lines has resulted in measurable waterlogging tolerance improvement under field conditions (Mano and Omori, 2007). Despite its aerenchyma advantage, maize lacks a Sub1A-equivalent transcriptional quiescence program and cannot strongly downregulate internode elongation under submergence, preventing it from conserving carbohydrate reserves for post-flood recovery. The antioxidant response to reoxygenation in maize is also less robust than in rice, particularly in ascorbate peroxidase and glutathione reductase activity, making it vulnerable to oxidative injury when floodwater recedes (Yeung et al., 2018). These deficiencies explain why maize yield losses under waterlogging remain substantial despite its aerenchyma advantage over wheat (Chen et al., 2026).

**Table 4** draws these threads together, comparing the four species side by side on the components that most directly determine translational relevance.

**Table 4:** Comparative hypoxia-tolerance components in wheat, maize, soybean, and barley.

Species	Aerenchyma	ADH/PDC induction	Antioxidant response	ERF-VII architecture
Wheat	Cortical porosity rises from about 2–3% to 10–15%, well below rice (15–30%) and below maize	*TaADH1/TaPDC* induced but more slowly and to a lower level than in rice, contributing to energy failure and acetaldehyde accumulation	Moderate and genotype-dependent; generally less effective than in rice	No *Sub1A* ortholog; ERF-VII stability follows the same oxygen/N-degron logic as *Arabidopsis*, without a dedicated submergence-tolerance allele
Maize	Cortical porosity rises from 3% to 20–30% within 48–96 h, comparable to rice, driven by strong ethylene sensitivity	Robust induction, but paired with high metabolic demand	Less robust than rice on reoxygenation, particularly for ascorbate peroxidase and glutathione reductase, leaving it vulnerable when floodwater recedes	Lacks a *Sub1A*-equivalent quiescence program; cannot downregulate elongation to conserve carbohydrate, so its aerenchyma advantage only partly offsets this gap
Soybean	Adventitious roots form with only modest aerenchyma compared with cereals	Present but comparatively modest; not a major focus of the literature to date	Variable; complicated by damage to nitrogen-fixing root nodules, a vulnerability cereals do not share	*GmERF3* is a tolerance-associated ERF-VII-like factor, though its architecture is less fully characterized than *Sub1A* in rice
Barley	Less extensive than in maize; barley is generally more waterlogging-sensitive than wheat despite sharing similar marginal soils	*HvADH1/HvPDC1* induced, but induction does not fully compensate for the oxygen deficit	Limited characterization relative to the other three species	*HvERF62* has recently been confirmed by CRISPR editing to affect waterlogging tolerance, the clearest ERF-VII functional link outside rice and *Arabidopsis*

Sources: Setter and Waters (2003); Drew et al. (2000); Mano and Omori (2007); Cornelious et al. (2005); Yeung et al. (2018); and Zhu et al. (2025). Values and responses are context- and genotype-dependent.

## 9 Impact on major crops

The mechanistic responses described in preceding sections translate into measurable yield penalties across all major crop species. The severity of loss depends on the crop’s inherent tolerance, the growth stage at which waterlogging occurs, and the duration of soil saturation.

### 9.1 Rice

Rice possesses greater inherent flood tolerance than other major cereals, supported by two primary adaptive strategies. Deepwater varieties use rapid internode elongation to remain above rising water, while most cultivated varieties rely on the *Sub1A*-mediated quiescence strategy described in Section 8.2. Marker-assisted breeding has transferred Sub1A into widely grown indica varieties such as *Swarna Sub1*, delivering yield advantages exceeding 1 t ha*−1* under flood conditions without penalty under normal growing conditions (Dar et al., 2018). Despite this progress, most conventional high-yielding rice varieties that lack the Sub1 locus tolerate complete submergence for only 3–4 days, and submergence beyond approximately 7 days causes severe damage or plant death in these varieties, with global assessments indicating flood-related rice production losses of approximately 4.3% annually (Li et al., 2025b).

### 9.2 Wheat

Wheat has low flood tolerance, with waterlogging affecting approximately 10–15 million hectares of wheat annually and causing yield losses of 25–30% worldwide (Pais et al., 2023). Flood damage is particularly severe during the reproductive phase. Waterlogging at anthesis causes the greatest yield reduction, including declines in leaf area and grain yield of up to 29%, while early tillering waterlogging typically reduces yield by 9–15% (Pais et al., 2023; Dolferus et al., 2011). Even brief waterlogging around booting or flowering can significantly reduce kernel number and weight (Pais et al., 2023). A genome-wide association study using 90K single nucleotide polymorphism (SNP) markers across 329 wheat genotypes has since mapped quantitative trait loci for two of the underlying adaptive traits, waterlogging-triggered adventitious roots and root cortical aerenchyma, with the strongest associations for both traits located on chromosome 4B (Xu et al., 2022).

### 9.3 Maize and soybean

Maize and soybean are both highly sensitive to excess water. In maize, eight days of waterlogging at vegetative stages reduced ear grain weight by approximately 60% in controlled trials, while ten days of flooding effectively killed the plants (Chen et al., 2026). Soybean yield losses can reach around 83% under severe flooding, with approximately 65% reduction when flooding occurs at the flowering stage for ten days (Gangana Gowdra et al., 2025). A global meta-analysis reported substantial crop-yield reductions under waterlogging, with severity depending on crop species, developmental stage, and stress duration (Tian et al., 2021). More recent QTL mapping in an F2:3 population derived from waterlogging-tolerant and -susceptible inbred lines identified 21 loci for waterlogging-responsive root and biomass traits, with major-effect QTL for root dry weight, diameter, and surface area clustering on chromosome 8 (Kumar et al., 2025).

### 9.4 Barley and other legumes

Barley is generally considered more waterlogging-sensitive than wheat, even though the two crops are often grown on the same marginal, poorly drained soils. Its aerenchyma formation is less extensive than in maize, and although ADH and PDC induction does occur, it does not fully compensate for the oxygen deficit. Genomic and physiological work continues to identify variation relevant to germination and early establishment under flooded soils, including how the timing of rainfall during seed development affects later flood tolerance (Gómez-Álvarez et al., 2025).

Grain legumes as a group remain understudied relative to cereals. Chickpea and lentil are highly sensitive to waterlogging and are typically excluded from flood-prone rotations rather than bred for tolerance, while field pea shows somewhat more variation between genotypes. Because legumes also host nitrogen-fixing rhizobia in root nodules, waterlogging disrupts symbiotic nitrogen fixation as well as the plant’s own metabolism, adding a pathway to yield loss that cereals do not share. This combination of higher sensitivity and a smaller research base means that, of the major crop groups covered in this review, grain legumes other than soybean have the least developed path toward waterlogging tolerance (Rane et al., 2021).

### 9.5 Yield penalties and critical stages

Across all major crops, flooding at or just before flowering consistently causes the greatest damage. Wheat suffers its greatest grain loss when waterlogged at anthesis (Pais et al., 2023), soybean loses approximately 65% of yield when flooded for ten days at flowering (Gangana Gowdra et al., 2025), and even rice experiences around 50% yield loss when submerged for more than seven days (Li et al., 2025b). Typical yield reductions range from approximately 30% to over 50% depending on the crop and duration of stress (Chen et al., 2026; Li et al., 2025b).

## 10 Screening and phenotyping approaches

Experiments designed to evaluate waterlogging tolerance use several complementary experimental setups, each with distinct advantages depending on the research objective. In pot-based tests, plants grow in pots or trays filled with soil that is flooded to approximately 110–120% of field capacity, depending on soil type and experimental protocol, and maintained for a defined period. Pots allow precise control of the growth medium and easy access to roots, facilitating measurement of traits such as adventitious root growth and aerenchyma formation (Langan et al., 2022). Field trials flood entire plots at specific growth stages, better simulating real agricultural conditions including soil variability and microbial activity, but require careful planning for irrigation sources, field levelling, and replication to ensure even flooding and avoid confounding factors. Hydroponic or agar-based hypoxia systems offer a third approach, where oxygen is removed by bubbling nitrogen gas to create root anoxia, allowing consistent and reproducible stress conditions and rapid screening of large germplasm collections, though they do not account for soil interactions (Langan et al., 2022).

In any experimental setup, stress imposition must be carefully calibrated. Factors including water level, oxygen concentration, flood duration, and recovery time are determined based on species and research objectives, with durations of 8–28 days being common in crop experiments. Pre-testing soil saturation and drainage through a pilot trial and then maintaining target water content either gravimetrically or with sensors throughout treatment is considered best practice (Langan et al., 2022).

Physiological indices are used to evaluate and compare tolerance across genotypes. A simple approach is to track survival rates or reductions in dry matter after a fixed flooding period. Stress indices such as the Stress Susceptibility Index are also calculated using the formula SSI = (1 − Ys/Yp)/D, where Ys and Yp are yields under stress and control conditions respectively, and D represents average stress intensity (Fischer and Maurer, 1978). Rapid physiological measurements are widely used for early stress detection. Chlorophyll fluorescence parameters such as F*v*/F*m* and photosystem II quantum yield decline quickly during hypoxia, and imaging fluorometers are commonly used as early indicators of stress before visible wilting occurs (Wu et al., 2024). Composite indices that combine survival with photosynthetic efficiency are particularly effective for ranking tolerant and susceptible lines across large germplasm collections.

Root traits are key phenotypes in waterlogging screening. Tolerant plants typically develop adventitious roots near the soil surface or above the waterline, alongside lysigenous aerenchyma in cortical tissue that promotes internal oxygen diffusion (Yamauchi et al., 2018). Researchers measure root porosity as the fraction of root volume containing gas, or calculate the percentage of aerenchyma area. Automated imaging tools including WinRHIZO scans and micro-computed tomography (micro-CT) are increasingly used to quantify adventitious root length, lateral root number, and gas space area with greater precision and throughput than manual methods. In summary, tolerant genotypes are identified through better survival and yield under flooding, alongside distinct root traits including greater adventitious rooting, higher cortical aerenchyma and porosity, and sustained photosynthetic function reflected in stable chlorophyll fluorescence parameters.

## 11 Management and mitigation strategies

### 11.1 Drainage systems

Installing surface and subsurface drains is the most direct method for removing excess water. Large-scale drainage approaches including surface furrows, mole drains, and buried tile drains lower the water table and significantly improve crop yields (Manik et al., 2019). Wheat losses due to waterlogging decreased substantially in southern Australia following the installation of subsurface tile drains. Surface spoon drains and interceptor banks are cost-effective options, with typical payback periods of 3–9 years. Drainage systems must be designed for local soil and climate conditions to ensure water can move efficiently into the drains (Manik et al., 2019).

### 11.2 Raised bed planting

Raising the seedbed above ground level keeps roots clear of the saturated zone. Permanent raised beds approximately 10–30 cm high and 2–3 m wide improve drainage and increase yields during flood events in irrigated and high-rainfall areas (Manik et al., 2019). In Australia and Mexico, raised beds boosted wheat and barley yields by 30–50% in waterlogged seasons. Beds also facilitate controlled traffic farming, which limits soil compaction, and allow quicker soil warming in cooler seasons. Growers should weigh the higher management costs of raised beds against their benefits when flood risk is low (Manik et al., 2019).

### 11.3 Soil amendments

Improving soil structure can accelerate drainage and reduce waterlogging severity. Organic amendments such as compost, biochar, and green manure increase soil porosity and microbial activity, aiding drainage. For sodic or compacted soils, gypsum or lime applied in soil slots can flocculate clay particles and reduce impermeable layers (Manik et al., 2019). Deep ripping or subsoiling breaks hardpans, and when combined with gypsum application, has improved drainage and subsequent crop growth (Manik et al., 2019).

### 11.4 Nutrient and water management

Waterlogged soils lose nitrogen and other nutrients through denitrification. Applying controlled-release fertilizers or foliar nutrients after flooding supports crop recovery (Manik et al., 2019). Slow-release nitrogen fertilizers have been shown to reduce nitrogen loss and support regrowth in flooded barley. Foliar applications of micronutrients including potassium and selenium can also help reduce oxidative stress during recovery. Fertilizer applications should be based on post-flood soil tests, with timing adjusted to occur after drainage to avoid further nutrient losses and imbalances (Shaviv, 2001; Pang et al., 2007).

### 11.5 Early warning and cultural controls

Timely planting during periods less prone to waterlogging can reduce exposure risk. Growing cover crops or flood-tolerant forages in flood-prone fields can also help, biologically draining the soil by absorbing excess water. Incorporating trees or deep-rooted perennials in wet areas works on the same principle, removing excess water through increased evapotranspiration. This approach, known as biodrainage, is well established as a low-cost complement to engineered drainage (Singh and Lal, 2018). Controlled traffic farming and raised furrows further limit compaction and improve long-term drainage capacity.

## 12 Climate change and future perspectives

### 12.1 Increasing extreme rainfall events

Climate change is intensifying the global hydrological cycle, increasing the frequency and severity of extreme rainfall events that cause agricultural waterlogging. Global climate models and recent observations confirm that heavy precipitation days and extreme downpours have increased in many regions, with a reported 7% per decade rise in heavy rainfall events since 1980 (Yan et al., 2024; IPCC, 2023). Areas already prone to waterlogging, including South Asia and sub-Saharan Africa, face greater exposure, while new regions may also experience flooding for the first time. Climate projections indicate that yield losses from waterlogging could double by 2080 under current emission trajectories, with low-lying paddy fields in Southeast Asia and river-irrigated plains in China particularly at risk if drainage infrastructure is not improved (see Section 1.1 for the uncertainty around these model-based estimates) (Tian et al., 2021; Liu et al., 2023). Even temperate agricultural regions could face late spring or early summer storms that saturate soils at critical growth stages such as booting or flowering, extending waterlogging stress well beyond the historical limits of most current cropping systems.

### 12.2 Need for climate-resilient crops

These climate trends sharpen the need for crops that can tolerate not just waterlogging in isolation but combinations of stresses that frequently co-occur, including heat, salinity, and drought. The combined effects of heat and waterlogging, or salinity and waterlogging, are typically more damaging than either stress alone. Future breeding programs must therefore target multi-stress resilience rather than single-stress tolerance. Varieties capable of withstanding temporary flooding, high temperatures, and occasional salt intrusion will be particularly important in coastal Asia and other flood-prone agricultural zones. Policymakers and researchers increasingly emphasize that without rapid adaptation, climate change could reverse previous gains in crop yields (Yan et al., 2024; IPCC, 2014). Meeting food security goals will require pairing new crop varieties with improved management strategies including better drainage infrastructure and early warning systems. Participatory breeding involving farmers from flood-prone areas will also be important for ensuring that developed solutions are matched to local flooding patterns and farming constraints.

### 12.3 Breeding and genome editing for flood tolerance

Developing flood-tolerant varieties is essential alongside agronomic management. Marker-assisted selection and genomic selection are now used to combine multiple tolerance traits in elite genetic backgrounds. *Sub1A* has already been introgressed into high-yielding rice varieties this way. Similar strategies are being pursued in wheat and maize, targeting QTLs for root porosity and fermentation enzyme activity (Bailey-Serres et al., 2012).

Genome editing offers a faster route to the same goal. CRISPR has been used to alter regulators of the hypoxia response, including both components that promote tolerance and those that suppress it. In rice and maize, researchers are editing adventitious-rooting pathways and ERF-VII factors directly. In maize, CRISPR/Cas9 knockout of the ERF-VII gene *ZmEREB180* confirmed its role directly: mutant seedlings showed reduced adventitious root formation and blunted induction of antioxidant genes under waterlogging relative to wild-type plants (Qi et al., 2025). CRISPR/Cas9 disruption of the *MsWOX13-2* gene in alfalfa improved chlorophyll retention and survival under waterlogging (Subedi et al., 2025). Targeting analogous hypoxia-response regulators in other crops is a promising route to flood resistance more broadly. A 2025 study extended this approach outside rice: using CRISPR/Cas9, it confirmed the ERF-VII gene *HvERF62* as a driver of waterlogging tolerance in barley. This moved the approach from candidate gene to confirmed function in a non-rice cereal (Zhu et al., 2025).

These approaches work best combined. Marker-assisted selection, CRISPR-based editing, speed breeding, and genomic prediction together offer the most complete strategy for tolerance improvement (Ahmed et al., 2013; Maher et al., 2020). Breeding programs in the coming decade will likely integrate all of these with physiological phenotyping. This way, flooding tolerance can improve alongside heat and other stresses at the same time.

### 12.4 Systems biology and predictive modeling

Advances in systems biology and crop modeling are essential tools for addressing climate-driven waterlogging risks. Multi-omics approaches including transcriptomics, proteomics, and metabolomics can uncover complex regulatory networks activated by multiple simultaneous stresses, helping identify key targets for tolerance improvement (Tyagi et al., 2023). High-throughput phenotyping technologies such as drone-based canopy imaging, hyperspectral reflectance sensing, and 3D root scanning allow rapid and precise screening of large germplasm collections under realistic flooding conditions. Crop simulation models such as Agricultural Production Systems Simulator (APSIM) already incorporate some flood dynamics, and merging these models with climate projections enhances risk assessment and varietal deployment decisions at the farm and regional levels. Molecular markers of stress resilience identified through systems biology can further inform decision-support tools for variety selection under predicted flood conditions.

## 13 Interaction with other abiotic stresses

### 13.1 Waterlogging and heat

High temperatures and flooding often coincide in summer, creating compound stress for crops. Waterlogging reduces oxygen availability in roots and lowers transpiration, while heat stress raises tissue temperature and promotes oxidative damage. Together these stresses substantially increase reactive oxygen species accumulation and impair photosynthesis (Li et al., 2025a). In Welsh onion, combining heat at 42 °C with waterlogging resulted in an approximately 88% drop in net photosynthesis, compared to 54% from heat alone and 26% from flooding alone (Li et al., 2025a). Similar negative interactions have been reported in rice and maize, where grain filling drops sharply under hot, waterlogged conditions. Yield losses from combined heat and flooding are consistently greater than from either stress individually.

To manage combined heat and waterlogging, farmers can adjust planting dates to avoid hot, rainy periods and ensure effective drainage to reduce root hypoxia during heat waves. Maintaining root zone aeration through subsurface drainage and using mulching to moderate soil temperature can also help. From a breeding perspective, enhancing both antioxidant defense and heat shock protein expression alongside root aeration traits offers a promising combined strategy. Overlapping stress signaling pathways involving ethylene and ABA interactions can be leveraged in marker-assisted or gene editing approaches targeting heat shock factor genes and fermentation enzymes together (Li et al., 2025a), building on evidence that the heat-inducible transcription factor HsfA2 enhances anoxia tolerance in Arabidopsis (Banti et al., 2010).

### 13.2 Waterlogging and salinity

Salinity and waterlogging frequently co-occur in coastal and irrigated lowlands. Plants facing saline flooding experience both ion toxicity from excess Na^+^ and Cl^−^ and oxygen shortage simultaneously. Waterlogging promotes soil reduction and releases toxic ions, while salinity hinders osmotic water uptake. In barley, the combination of 200 mM NaCl and waterlogging reduced biomass by half and doubled leaf Na^+^ compared to salinity alone, while leaf K^+^ decreased by approximately 40% (Zeng et al., 2013). Chlorophyll content and photosystem II efficiency also declined more severely under combined stress than under either treatment individually. These synergistic effects are closely linked to redox-driven Fe^2+^ and Mn^2+^ toxicities under low oxygen conditions. Field reports from rice paddies affected by seawater intrusion indicate that combined waterlogging and salinity frequently causes greater yield reductions than either stress alone, sometimes approaching total crop failure in sensitive varieties. Wheat grown on sodic, waterlogged soils is reported to suffer substantial yield loss without appropriate management, sometimes cited as high as 50–80%, though well-controlled quantitative estimates specific to this combined stress remain scarce.

Managing combined salinity and waterlogging requires addressing both stresses simultaneously. This may involve installing drainage systems to leach salts, applying gypsum to displace Na^+^, and selecting salt-tolerant rootstocks or companion crops. In breeding, combining tolerance to both stresses through marker-assisted selection offers a practical approach. Research in barley and rice is isolating loci that improve K^+^/Na^+^ selectivity under low oxygen conditions, and genomic selection models targeting both salt and hypoxia tolerance traits can accelerate the development of varieties resilient to both stresses.

### 13.3 Combined stress physiology

When plants encounter multiple simultaneous stresses such as flooding, heat, and salinity, their responses are often complex and unpredictable. Combined stresses frequently produce synergistic effects that create damage patterns not seen under any single stress. Flooding combined with high temperatures typically generates more hydrogen peroxide and greater lipid peroxidation than either stress alone (Li et al., 2025a). Hormonal signaling pathways involving ethylene, ABA, and auxin also respond in complex and sometimes antagonistic ways under dual stresses, with ROS-scavenging enzymes and heat shock proteins being co-activated at a higher metabolic cost to growth. In barley, the salt and flooding combination caused more severe chlorosis and stunted growth than either treatment alone (Zeng et al., 2013). In rice and maize, flooding during flowering combined with heat is known to impair grain filling, though the two stresses do not necessarily compound in a simple additive way. A recent review of the limited literature on this specific combination found antagonistic interactions, where one stress partly buffers the damage from the other, to be more common than synergistic worst-case outcomes (Ploschuk et al., 2025). These findings underline the importance of developing both management and breeding strategies specifically designed for combined stress scenarios. Whether this is achievable at all is itself a live question: a recent assessment of multi-stress breeding across crops concluded that it is feasible in principle, but only with far closer collaboration between breeders, physiologists, and policymakers than currently exists (Khazaei et al., 2026).

Management strategies for combined stress should focus on building overall system resilience. Maintaining healthy soil organic matter and effective drainage provides protection against both flooding and heat events. Cover crops and mulches can simultaneously moderate soil temperature and improve water absorption. From a breeding perspective, integrating low-oxygen tolerance through *Sub1A* or fermentation enzyme genes with traits that support heat avoidance such as early maturity or adjusted phenology could produce varieties capable of avoiding peak combined stress periods (Yan et al., 2024). **Table 5** summarizes crop-specific vulnerabilities, physiological markers, and recommended management and breeding strategies for combined flood–heat and flood–salinity stress in rice, wheat, maize, and soybean.

**Table 5:** Comparison of combined-stress effects and mitigation strategies for major crops.

Crop	Main vulnerabilities	Key physiological markers	Recommended management	Breeding/genomic strategies
Rice	Flooding causes root anoxia; heat exacerbates leaf damage; salinity adds ion toxicity	Chlorophyll fluorescence decline; root aerenchyma formation; Na^+^/K^+^ ratio	Raised beds or good drainage; adjust planting to avoid hot floods; salt leaching	Introgress *Sub1A* and heat-tolerance genes; pyramid flood and salt tolerance *QTL*; CRISPR editing
Wheat	Sensitive at flowering; combined waterlogging and heat/salt reduce grain set	Spikelet sterility; leaf water potential; stomatal conductance	Soil drainage improvements; avoid late-season irrigation; K fertilization for salt	Select for early-maturing flood-tolerant varieties; marker-assisted selection (MAS) for known *QTL*; genomic selection
Maize	Flooding impedes root respiration; heat increases transpiration; sensitive early	Aerenchyma proportion; leaf rolling; ROS levels	Raised beds/contours; ensure rapid field drainage; shade nets in nursery	Breed for root aeration and heat shock protein expression; identify salt-blocking root traits
Soybean	Hypoxia reduces nodulation; heat impairs flowering; salt disrupts osmotic balance	Nodule viability; chlorophyll content; leaf water content	Use tolerant rootstocks; controlled irrigation scheduling; organic soil amendments	Integrate traits for nodule resilience; MAS for combined stress *QTL*; CRISPR on regulatory genes

Responses are context-dependent and vary with crop genotype, developmental stage, water depth and duration, salinity level, temperature regime, and post-stress recovery conditions.

## 14 Knowledge gaps and future directions

### 14.1 Mechanisms of long-term stress memory

Plants can retain memory of past stress experiences, modifying their responses to subsequent events. However, the molecular basis of stress memory specifically related to waterlogging remains poorly characterized. Key unresolved questions include how hypoxia-related chromatin marks form, how they are maintained through cell divisions, how they are reset between flooding events, and whether they can be transmitted across generations. Time-series transcriptomic and epigenomic studies following repeated waterlogging cycles could reveal how memory marks persist and which signals encode hypoxia experiences. Answers to these questions could open new approaches to breeding or chemical priming strategies that improve crop resilience to intermittent flooding. A recently proposed integrative framework for abiotic stress priming and memory, which distinguishes somatic, intergenerational, and transgenerational timescales, offers a useful conceptual starting point for organizing this work in a flooding-specific context (Aswathi et al., 2025).

### 14.2 Root architecture engineering

Root structure is essential for waterlogging survival, yet root traits remain underutilized in most crop breeding programs. In rice, three traits have been identified as critical for flood survival: cortical aerenchyma, a barrier to radial oxygen loss, and shoot-borne adventitious roots. While the genetic control of these traits is well characterized in rice, their counterparts in wheat, maize, and soybean are not well understood, limiting the transfer of root engineering strategies beyond the model crop. High-throughput root phenotyping under low oxygen conditions is needed, including tools such as X-ray tomography and automated root image analysis to measure tissue porosity, stele-to-cortex ratios, and adventitious root number during flooding. Targeted genome editing using CRISPR/Cas approaches offers a way to introduce or modify root traits rapidly without lengthy breeding cycles, for example by editing negative regulators of aerenchyma formation or adventitious rooting to accelerate these adaptive responses in elite varieties of non-rice cereals.

Progress on the imaging side is already underway: a 2025 review of high-throughput root phenotyping catalogues how X-ray computed tomography, magnetic resonance imaging, and machine learning-based image segmentation are being applied to resolve internal traits such as aerenchyma and stele-to-cortex ratio non-destructively and at scale, including in cereal root systems (Chandnani and Soolanayakanahally, 2025). An X-ray computed tomography method has already been used to track waterlogging-induced aerenchyma formation in barley roots over time without destructive sectioning, detecting air-filled tissue as early as nine days after waterlogging began; extending this kind of longitudinal, non-destructive imaging to wheat, maize, and soybean would help close the phenotyping gap described above (Kehoe et al., 2022).

### 14.3 Epigenetic modifications

Epigenetic regulation is an increasingly recognized dimension of stress adaptation, but its role in waterlogging tolerance has not been studied comprehensively. Research in *Arabidopsis* and several crop species has shown that DNA methylation and histone modifications can prime stress-responsive genes for faster reactivation. However, comprehensive methylome and histone modification maps across waterlogged tolerant and sensitive genotypes are largely lacking, and it remains unclear how stable these marks are across cell divisions or generations. Future studies should conduct methylome and histone chromatin immunoprecipitation (ChIP) profiling to identify epigenetic markers of waterlogging tolerance and determine whether modifying specific DNA methyltransferases or histone demethylases can improve flood resilience. The potential for chemical priming agents to induce lasting epigenetic changes that improve tolerance also warrants systematic investigation in the context of waterlogging. Beyond profiling existing marks, targeted epigenome-editing tools capable of writing or erasing specific DNA methylation states are now being extended from *Arabidopsis* into crop species, offering a more direct way to test whether a given methylation state is actually causal for flood tolerance rather than merely correlated with it (Wang and Bart, 2025).

### 14.4 Translational research from model plants to crops

A substantial body of mechanistic knowledge about hypoxia signaling has been generated in model species including *Arabidopsis*, rice, and maize, but translation of these findings into practical improvements in other major crops remains slow. Key oxygen-sensing pathways including the N-degron mechanism and ERF-VII regulatory network are conserved across species, and manipulating these pathways has improved flood tolerance in barley, wheat, and soybean in experimental settings. However, most crop improvement programs do not routinely incorporate these targets, and discoveries from model plants frequently remain confined to laboratory studies. Establishing cross-species translation pipelines that identify orthologous genes, validate them in crop genetic backgrounds, and test their effects under realistic field flooding conditions is a priority need. Advances in CRISPR libraries, speed breeding, and genomic selection can accelerate this process. Effective translation will require coordinated teams of molecular biologists, crop physiologists, breeders, and agronomists working together to adapt mechanistic insights into field-ready solutions for diverse crops and environments. The barley and maize CRISPR examples described in Section 12.3 show this translation already starting to happen, with ERF-VII biology first defined in *Arabidopsis* and rice now validated by gene editing directly in two non-model cereals.

## 15 Conclusion

Waterlogging and hypoxia represent an escalating threat to global food security, affecting 12–27% of cultivated land annually and causing average yield losses of 20–33% across major crops under prolonged flooding (Tian et al., 2021; Li et al., 2025b). This review has traced a mechanistic path from oxygen sensing through the N-degron pathway, through fermentative biochemistry and aerenchyma development, to the hormonal and genetic architecture of flood tolerance across crop species (Voesenek and Bailey-Serres, 2015). These elements function as an integrated system, not independent pathways, all serving the same goal of cellular survival during flooding. This is best demonstrated by Sub1 rice, where introgression of a single ERF-VII allele delivered 14–18 additional days of submergence survival and yield gains of 1.0–2.5 t ha*−1* in flood-prone regions of South and Southeast Asia, with no yield penalty under favorable conditions (Ismail et al., 2013).

Several important advances since 2019 have refined the classical N-degron model, from NO-mediated stabilization and mRNA-level oxygen sensing to single cell-type regulatory network mapping and recognition of reoxygenation as a distinct stress phase in its own right (Hartman et al., 2019; Weits et al., 2019; Reynoso et al., 2019; Yeung et al., 2018).

Significant knowledge gaps remain: the molecular basis of flooding stress memory, genetic control of root traits in non-rice cereals, comprehensive epigenomic maps, and pipelines for translating mechanistic insights into field-ready improvements. Closing these will require collaboration across molecular biology, physiology, genomics, agronomy, and socioeconomics.

Looking ahead, four complementary strategies offer the most promising paths to flood-resilient agriculture. First, multi-omics QTL mapping integrating transcriptomic, proteomic, metabolomic, and epigenomic variation with field phenotypes can uncover new tolerance alleles in wheat, maize, and soybean. Second, microbiome engineering with ACC-deaminase-producing PGPR offers a genotype-independent strategy across diverse crop systems (Glick, 2014). Third, systematic study of epigenetic stress memory and transgenerational priming could open heritable routes to flood resilience. Fourth, combining OsTPP7 and reoxygenation-tolerance QTLs with either Sub1A or SK1/SK2, selected according to the flooding regime of the target environment, offers a practical near-term path to broad tolerance in rice, with similar multi-gene strategies needed for wheat and maize (Bailey-Serres et al., 2019). Integrating these approaches is both the central challenge and the greatest opportunity for the field in the coming decade.
